# Coupled molybdenum carbide and reduced graphene oxide electrocatalysts for efficient hydrogen evolution

**DOI:** 10.1038/ncomms11204

**Published:** 2016-04-01

**Authors:** Ji-Sen Li, Yu Wang, Chun-Hui Liu, Shun-Li Li, Yu-Guang Wang, Long-Zhang Dong, Zhi-Hui Dai, Ya-Fei Li, Ya-Qian Lan

**Affiliations:** 1 Jiangsu Key Laboratory of Biofunctional Materials, College of Chemistry and Materials Science, Nanjing Normal University, Nanjing 210023, China; 2 Key Laboratory of Inorganic Chemistry in Universities of Shandong, Department of Chemistry and Chemical Engineering, Jining University, Qufu, Shandong 273155, China

## Abstract

Electrochemical water splitting is one of the most economical and sustainable methods for large-scale hydrogen production. However, the development of low-cost and earth-abundant non-noble-metal catalysts for the hydrogen evolution reaction remains a challenge. Here we report a two-dimensional coupled hybrid of molybdenum carbide and reduced graphene oxide with a ternary polyoxometalate-polypyrrole/reduced graphene oxide nanocomposite as a precursor. The hybrid exhibits outstanding electrocatalytic activity for the hydrogen evolution reaction and excellent stability in acidic media, which is, to the best of our knowledge, the best among these reported non-noble-metal catalysts. Theoretical calculations on the basis of density functional theory reveal that the active sites for hydrogen evolution stem from the pyridinic nitrogens, as well as the carbon atoms, in the graphene. In a proof-of-concept trial, an electrocatalyst for hydrogen evolution is fabricated, which may open new avenues for the design of nanomaterials utilizing POMs/conducting polymer/reduced-graphene oxide nanocomposites.

## Abstract (web summary)

The development of low-cost and earth-abundant non-noble-metal catalysts for the hydrogen evolution reaction remains a challenge. Here, the authors report and evaluate a catalyst based on a two-dimensional coupled hybrid of molybdenum carbide and reduced graphene oxide.

## Graphical abstract

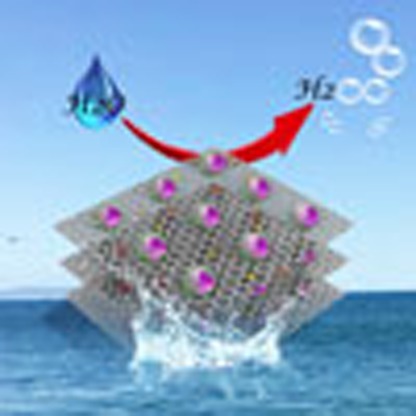

To address the energy crisis and ameliorate environmental contamination, researchers have devoted considerable attention to hydrogen as promising alternative to fossil fuels. Electrochemical water splitting to produce hydrogen, or the hydrogen evolution reaction (HER), is the most economical and sustainable method for large-scale hydrogen production. Achieving this goal requires inexpensive electrocatalysts with high efficiency for the HER^1^,^2^. Although the best electrocatalysts are Pt or Pt-based materials, their high cost and low abundance substantially hamper their large-scale utilization^3^,^4^,^5^. Thus, the development of low-cost and earth-abundant non-noble-metal catalysts to replace Pt is an important and urgently needed for practical applications.

Because of their Pt-like catalytic behaviours^6^, Mo-based compounds, such as Mo_2_C^7^,^8^,^9^,^10^, MoN^11^,^12^,^13^, MoS_2_ (refs 14, 15, 16, 17), and others^18^,^19^,^20^ have attracted substantial interest as a new class of electrocatalysts. To further enhance the HER activity, Mo-based compounds have been anchored onto conductive supports, such as carbon nanosheets (NSs)^21^,^22^,^23^ and carbon nanotubes (CNTs)^11^,^24^,^25^, which not only prevent Mo-based compounds from aggregating but also increase the dispersion of active sites. Among these conductive supports, reduced graphene oxide (RGO), particularly nitrogen (N)-doped RGO, has garnered much attention because of its excellent electron transport properties and chemical stability^26^,^27^. Therefore, RGO-supported Mo-based compounds appear to be highly active and stable electrocatalysts^11^,^25^,^28^,^29^,^30^. However, carbonization at high-reaction temperature during synthesis procedures leads to the sintering and aggregation of Mo-based-compound nanoparticles (NPs), thus reducing their number of exposed active sites and their specific surface area^8^,^19^. In addition, due to its strong π-stacking and hydrophobic interactions, RGO NSs usually aggregate, which hinders their practical application^31^,^32^. Preventing the RGO from re-stacking and the Mo-based compound NPs from aggregating during the synthesis of a porous uniform thin layer RGO-supported Mo-based electrocatalysts is critical to enhancing their catalytic performance.

We developed a new approach to integrate polyoxometalates (POMs) and pyrrole (Py) on graphene substrates via a “one-pot” method to obtain ternary POMs–polypyrrole/RGO (POMs–PPy/RGO) nanohybrid sheets with a uniform distribution. As an important family of transition-metal oxide clusters with excellent redox features^33^,^34^, POMs provided an essential oxidizing medium for the oxidative polymerization of Py^35^, and the POMs finally were converted into “heteropoly blue”^36^. Heteropoly blue can be used as a highly localized reducing agent and can further react with graphene oxide (GO) to restore the original POMs. With the polymerization of the Py monomers, POMs were dispersed into the PPy framework. Meanwhile, RGO was homogeneously dispersed and segregated by both the POMs and PPy during the synthesis of POMs–PPy/RGO. Thus, RGO-supported Mo-based catalysts prepared with POMs–PPy/RGO as a precursor may efficiently hinder the Mo sources and graphene from aggregating during the process of forming the RGO-supported NPs. To the best of our knowledge, reports on POMs, PPy and RGO ternary hybrids by a green and one-pot redox relay reaction are rare. More importantly, the coupled hybrid with both Mo_2_C and RGO has not been previously prepared with a ternary hybrid as the precursor.

In this work, we carefully design and fabricate a two-dimensional (2D) coupled hybrid consisting of Mo_2_C encapsulated by N, phosphorus (P)-codoped carbon shells and N, P-codoped RGO (denoted as Mo_2_C@NPC/NPRGO) using a PMo_12_ (H_3_PMo_12_O_40_)–PPy/RGO nanocomposite as the precursor. Notably, the entire polymerization and the reductive reactions are triggered by PMo_12_ without any additional oxidants or reductants, leading to a synthetic process that is green, efficient and economical. PPy was used as both the carbon and nitrogen sources as well as the reducing agent for GO. Three main advantages of this method are attributed to the Mo_2_C@NPC/NPRGO hybrid: (1) due to the unique structure of PMo_12_–PPy/RGO, the Mo_2_C NPs are nanosized and uniformly embedded in the carbon matrix without aggregation; (2) the Mo_2_C NPs are coated with carbon shells, which effectively prevent Mo_2_C NPs from aggregating or oxidizing and impart them with fast electron transfer ability; and (3) owing to the heteroatom dopants (N, P), a large number of active sites are exposed. Overall, taking advantage of the synergistic catalytic effects, the Mo_2_C@NPC/NPRGO catalyst exhibits excellent electrocatalytic activity for the HER, with a low onset overpotential of 0 mV (vs reversible hydrogen electrode (RHE)), a small Tafel slope of 33.6 mV dec^−1^, and excellent stability in acidic media. Its HER catalytic activity, which is comparable to that of commercial Pt–C catalyst, even superior to those of the best reported non-noble-metal catalysts. In addition, we further investigate the nature of catalytically active sites for the HER using density functional theory (DFT). This approach provides a perspective for designing 2D nanohybrids with transition-metal carbides and RGO as HER catalysts.

## Results

### Catalyst synthesis and characterization

**Figure 1:**
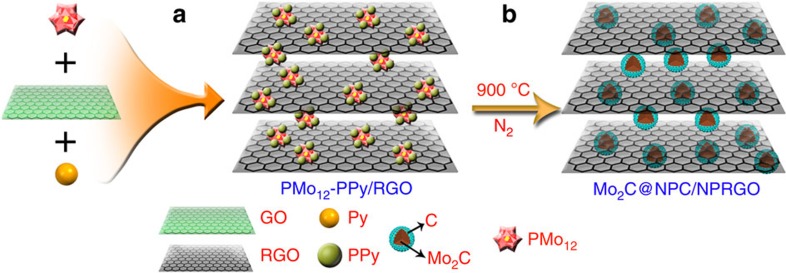
Schematic illustration of the synthetic process of Mo_2_C@NPC/NPRGO. (**a**) Synthesis of PMo_12_–PPy/RGO via a green one-pot redox relay reaction. (**b**) Formation of Mo_2_C@NPC/NPRGO after carbonizing at 900 °C.

Mo_2_C@NPC/NPRGO was synthesized as follows: (1) the PMo_12_–PPy/RGO nanocomposite was synthesized via a green one-pot redox relay reaction. The nanocomposite was then carbonized under a flow of ultrapure N_2_ at 900 °C for 2 h at a heating rate of 5 °C min^−1^. Finally, the obtained samples were acid etched in 0.5 M H_2_SO_4_ for 24 h with continuous agitation at 80 °C to remove unstable and inactive species. The etched samples were thoroughly washed with de-ionized water until the pH of the wash water was neutral (Fig. 1).

**Figure 2:**
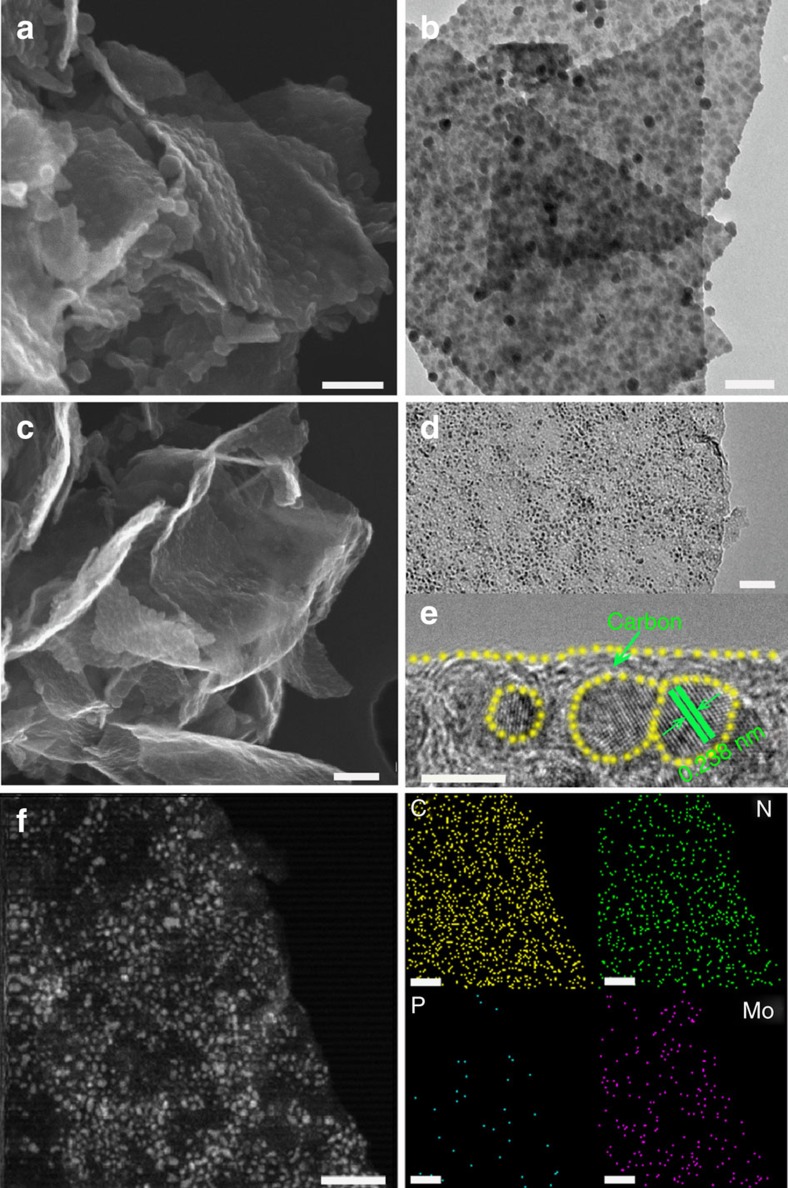
Characterization of the PMo_12_–PPy/RGO and Mo_2_C@NPC/NPRGO hybrids. (**a**) SEM and (**b**) TEM images of PMo_12_–PPy/RGO. (**c**) SEM, (**d**) TEM, (**e**) HRTEM and (**f**) STEM images and EDX elemental mapping of C, N, P and Mo of Mo_2_C@NPC/NPRGO. Scale bar: **a**,**b**,**c** (200 nm); **d** (100 nm); **e** (5 nm) and **f** (50 nm).

Figure 2a shows a scanning electron microscopy (SEM) image of PMo_12_–PPy/RGO. The rough surfaces and wrinkled edges on the sheet-like structures were due to the intercalation and polymerization of Py. A transmission electron microscopy (TEM) image of PMo_12_–PPy/RGO revealed that a large amount of PPy/PMo_12_ NPs were homogeneously coated onto the RGO NSs and that voids were present (Fig. 2b). As evident in Fig. 2c and d, the morphologies of Mo_2_C@NPC/NPRGO were similar to that of PMo_12_–PPy/RGO after carbonization. The nanosized Mo_2_C NPs with diameters of ∼2–5 nm were uniformly decorated on the RGO sheets at a high density, which was attributed to the distinct porous structure of PMo_12_–PPy/RGO. The high-resolution TEM (HRTEM) image exhibited clear lattice fringes with an interplanar distance of 0.238 nm, corresponding to the (111) planes of Mo_2_C (Fig. 2e)^37^. Notably, the Mo_2_C NPs were embedded in the carbon shells, which can efficiently prevent the aggregation and/or excessive growth of Mo_2_C NPs^22^. Figure 2f shows the scanning TEM (STEM) and corresponding energy dispersive X-ray spectroscopy (EDX) elemental mapping images, which confirmed that C, Mo, N and P were distributed on the Mo_2_C@NPC/NPRGO surface, consistent with the EDX spectrum (Supplementary Fig. 1). These results confirm the successful synthesis of the Mo_2_C@NPC/NPRGO nanocomposite.

For comparison, the nanohybrid of Mo_2_C encapsulated by N, P-codoped carbon (defined as Mo_2_C@NPC) was also synthesized through a similar preparation procedure without GO. Supplementary Fig. 2a shows aggregation of PPy/PMo_12_ NPs. Supplementary Fig. 2b and c reveals that Mo_2_C NPs tended to agglomerate during the heat treatment to form large NPs, which decreased the exposed active surface. Supplementary Fig. 2d demonstrates the STEM and EDX elemental mapping images of Mo_2_C@NPC. These data verified that the Mo_2_C@NPC material contained C, N, P and Mo elements, consistent with the EDX results (Supplementary Fig. 1b). Hence, these results sufficiently confirm that the presence of GO plays an important role in the generation of highly dispersed and nanosized Mo_2_C NPs.

Supplementary Fig. 3 shows the powder X-ray diffraction patterns of Mo_2_C@NPC and Mo_2_C@NPC/NPRGO. The broad peak at ∼25° was ascribed to carbon^38^,^39^. The other peaks located at 37.9, 43.7, 61.6 and 75.6° were indexed to the (111), (200), (220) and (311) planes of Mo_2_C (JCPDS, No. 15-0457), respectively; these peaks were broad and exhibited low intensity because of the smaller crystallites of Mo_2_C or Mo_2_C coated with amorphous carbon^21^,^40^,^41^. Beside, the degrees of graphitization of the two catalysts were analyzed by Raman spectra (Supplementary Fig. 4). As is well-known, the ratio between the D (1,350 cm^−1^) and G band (1,580 cm^−1^) intensities (*I*_D_/*I*_G_) is an important criterion to judge the degree of the graphitization^9^,^28^. Compared to Mo_2_C@NPC, the *I*_D_/*I*_G_ of Mo_2_C@NPC/NPRGO is higher, implying that more defects formed on the RGO sheets, thus favoring the accessibility of more active sites and enhancing the electrocatalytic performance. The Brunauer–Emmett–Teller (BET) surface areas of Mo_2_C@NPC and Mo_2_C@NPC/NPRGO calculated by the N_2_ sorption isotherms were 55 and 190 m^2^ g^−1^, respectively (Supplementary Fig. 5a). Mo_2_C@NPC showed a microporous structure, with pore sizes mainly in the range from 1 to 2 nm (Supplementary Fig. 5b), whereas the corresponding pore size distribution of Mo_2_C@NPC/NPRGO was mainly concentrated in the range from 1 to 10 nm, which was characteristic of a microporous and mesoporous structure (Supplementary Fig. 5c). Overall, the large surface area and enriched porous structures efficiently facilitate electrolyte penetration and charge transfer^9^.

**Figure 3:**
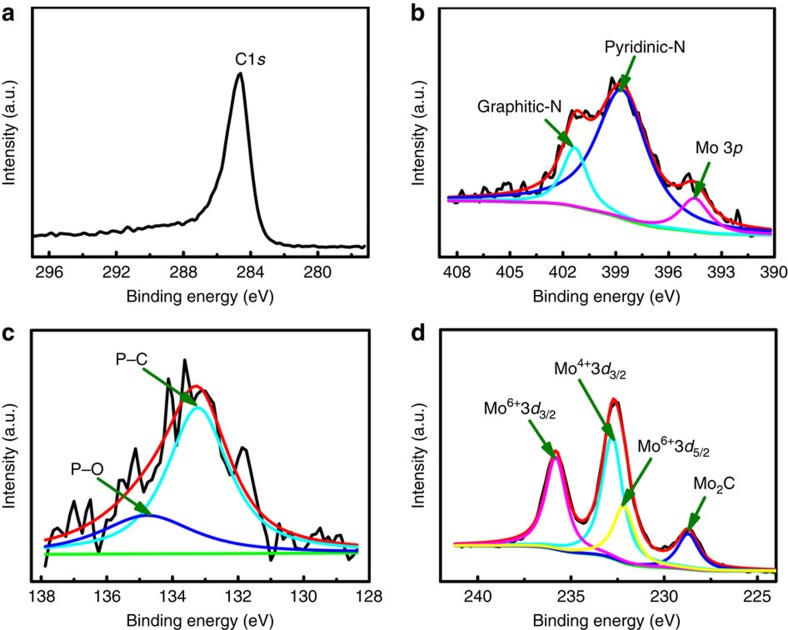
Compositional characterization of the Mo_2_C@NPC/NPRGO. XPS high-resolution scans of (**a**) C 1*s*, (**b**) N 1*s*, (**c**) P 2*p* and (**d**) Mo 3*d* electrons of Mo_2_C@NPC/NPRGO.

X-ray photoelectron spectroscopy (XPS) analyses of Mo_2_C@NPC/NPRGO catalysts were carried out to elucidate their valence states and compositions. As observed, the XPS spectrum of Mo_2_C@NPC/NPRGO (Supplementary Fig. 6) indicated the presence of C, N, O, P and Mo in the catalyst. The deconvoluted C1*s* spectrum is shown in Fig. 3a, and the main peak at 284.6 eV implies that the graphite carbon is the majority species^22^. The deconvolution of N1*s* energy level signals for Mo_2_C@NPC/NPRGO revealed the peaks at 398.6 and 401.3 eV, which were assigned to pyridinic and graphitic N (Fig. 3b), respectively^21^,^27^. From Fig. 3c, it can be seen that the P2*p* peaks at about 133.5, and 134.8 eV were attributed to P–C and P–O bonding, respectively^18^,^28^. Besides, the high-resolution Mo 3*d* XPS revealed that the peak at 228.8 eV was attributable to Mo^2+^, stemming from Mo_2_C. In parallel, as a consequence of surface oxidation, the peaks at 232.05 and 235.2 were attributable to MoO_3_ and that at 232.7 eV was assignable to MoO_2_ (refs 8, 21); both of these species are inactive toward the HER (Fig. 3d). For comparison, Mo_2_C@NPC is shown in Supplementary Fig. 7. All of these data were similar to those for Mo_2_C@NPC/NPRGO. The corresponding atomic percentages of the different catalysts measured by XPS are listed in Supplementary Table 1.

### Electrocatalytic HER performance

**Figure 4:**
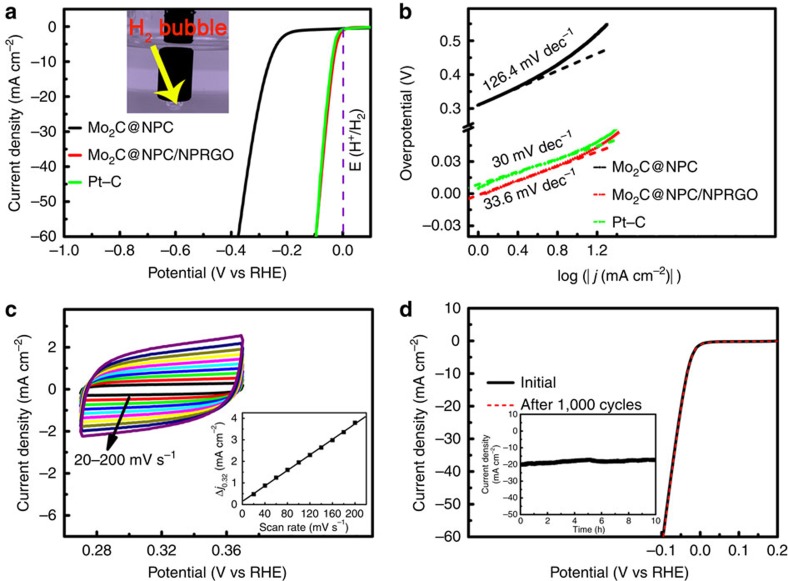
HER activity characterization. (**a**,**b**) Polarization curves and Tafe plots of Mo_2_C@NPC, Mo_2_C@NPC/NPRGO and Pt–C. (inset: the production of H_2_ bubbles on the surface of Mo_2_C@NPC/NPRGO). (**c**) CVs of Mo_2_C@NPC/NPRGO with different rates from 20 to 200 mV s^−1^. Inset: The capacitive current at 0.32 V as a function of scan rate for Mo_2_C@NPC/NPRGO. (**d**) Polarization curves of Mo_2_C@NPC/NPRGO initially and after 1,000 CV cycles. Inset: Time-dependent current density curve of Mo_2_C@NPC/NPRGO under a static overpotential of 48 mV for 10 h.

A three-electrode system was adopted to evaluate the electrocatalytic activities of Mo_2_C@NPC/NPRGO toward the HER in 0.5 M H_2_SO_4_ at 100 mV s^−1^. For comparison, Mo_2_C@NPC and commercial Pt–C (20 wt% Pt on carbon black from Johnson Matthey) were also assessed. The corresponding polarization curves without IR compensation are shown in Fig. 4a. All potentials in this work are reported vs RHE. As expected, the commercial Pt–C displayed the highest electrocatalytic activity, with an onset overpotential of nearly zero^30^. The Mo_2_C@NPC catalyst exhibited far inferior HER activity. Impressively, Mo_2_C@NPC/NPRGO exhibited the lowest onset overpotential of 0 mV, approaching the performance of commercial Pt–C. Moreover, it was clearly observed that the cathodic current rose sharply with more negative potentials. Generally, the potential value for a current density of 10 mA cm^−2^ is an important reference because solar-light-coupled HER apparatuses usually operate at 10–20 mA cm^−2^ under standard conditions (1 sun, AM 1.5)^4^. To achieve this current density, Mo_2_C@NPC requires an overpotential of 260 mV. Strikingly, Mo_2_C@NPC/NPRGO required only ∼34 mV to achieve a 10 mA cm^−2^ current density, even superior to commercial Pt–C (40 mV) (Table 1). To the best of our knowledge, this overpotential is superior to those of all previously reported non-noble-metal electrocatalysts for the HER, such as MoS_2_/CoSe_2_ (ref. 15), MoO_2_ (ref. 18), Mo_2_C/CNT^24^ and CoNi@NC^40^ (Supplementary Table 2).

To elucidate the HER mechanism, Tafel Plots were fitted to Tafel equation (that is, *η*=*b* log (*j*) + *a*, where *b* is the Tafel slope, and *j* is the current density), as shown Fig. 4b. The Tafel slope of commercial Pt–C was ∼30 mV dec^−1^, which was in agreement with the reported value, thus supporting the validity of our electrochemical measurements^30^. The Tafel slope of Mo_2_C@NPC/NPRGO was 33.6 mV dec^−1^, which indicated higher performance than that of Mo_2_C@NPC (126.4 mV dec^−1^). Meanwhile, the Tafel slope of Mo_2_C@NPC/NPRGO suggested that hydrogen evolution on the Mo_2_C@NPC/NPRGO electrode probably proceeds via a Volmer–Tafel mechanism, where the recombination is the rate-limiting step^17^. The exchange current density (*j*_0_) was extrapolated from the Tafel plots. Notably, Mo_2_C@NPC/NPRGO displayed the largest *j*_0_ of 1.9 × 10^−3 ^A cm^−2^, which was nearly three times larger than the *j*_0_ of Pt–C (0.39 × 10^−3 ^A cm^−2^) (Table 1) and was substantially greater than those of other recently reported non-noble-metal catalysts (Supplementary Table 2). This performance of Mo_2_C@NPC/NPRGO demonstrates favourable HER kinetics at the Mo_2_C@NPC/NPRGO/electrolyte interface.

The electrochemical double-layer capacitance (EDLC, *C*_dll_) was measured to investigate the electrochemically active surface area. Cyclic voltammetry (CV) was performed in the region from 0.27 to 0.37 V at rates varying from 20 to 200 mV s^−1^ (Fig. 4c and Supplementary Fig. 8). The *C*_dll_ of Mo_2_C@NPC/NPRGO (17.9 mF cm^−2^) was ∼195 times larger than that of Mo_2_C@NPC (0.092 mF cm^−2^). Thus, the large *j*_0_ value of Mo_2_C@NPC/NPRGO may benefit from both its large BET surface area and its large EDLC.

To gain further insight into the electrocatalytic activity of Mo_2_C@NPC/NPRGO for the HER, we performed electrochemical impedance spectroscopy (EIS). The Nyquist plots of the EIS responses are shown in Supplementary Fig. 9. Compared with the Nyquist plot of Mo_2_C@NPC, that of Mo_2_C@NPC/NPRGO showed a much smaller semicircle, suggesting that Mo_2_C@NPC/NPRGO has lower impedance. This result proves that the catalyst affords markedly faster HER kinetics due to the presence of the RGO support.

Long-term stability is also critical for HER catalysts. To probe the durability of the Mo_2_C@NPC/NPRGO catalyst, continuous CV was performed between −0.2 and 0.2 V at a 100 mV s^−1^ scan rate in 0.5 M H_2_SO_4_ solution (Fig. 4d). As observed, the polarization curve for Mo_2_C@NPC/NPRGO remained almost the same after 1,000 cycles. In addition, the durability of Mo_2_C@NPC/NPRGO was also examined by electrolysis at a static overpotential of 48 mV. The inset of Fig. 4d shows that the current density experienced a negligible loss at ∼20 mA cm^−2^ for 10 h. For comparison, the durability of the Mo_2_C@NPC catalyst was examined by the same methods (Supplementary Fig. 10). This is reconfirming that Mo_2_C@NPC and Mo_2_C@NPC/NPRGO are stable electrocatalysts in acidic solutions.

**Figure 5:**
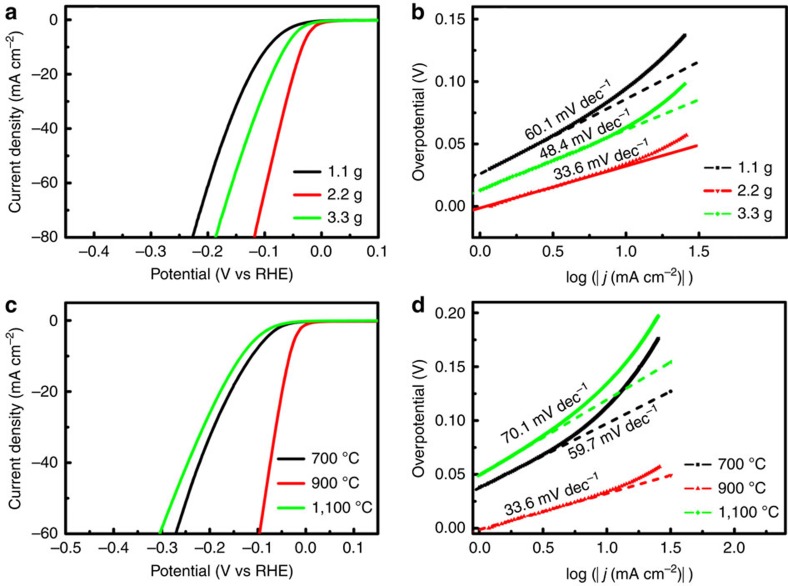
Comparison of the HER performance of different electrocatalysts. (**a**,**b**) Polarization curves and Tafe plots of Mo_2_C@NPC/NPRGO with different mass of PMo_12_ (1.1, 2.2 and 3.3 g). (**c**,**d**) Polarization curves and Tafe plots of Mo_2_C@NPC/NPRGO (2.2 g) at different carbonization temperature.

In control experiments, we investigated the effect of the PMo_12_ content on electrocatalytic performance. Two other catalysts with different PMo_12_ contents (1.1 and 3.3 g) were synthesized (denoted as Mo_2_C@NPC/NPRGO-1.1 and Mo_2_C@NPC/NPRGO-3.3). The morphology, structure and composition of these two catalysts were studied by SEM, TEM, HRTEM, STEM, EDX, elemental mapping, powder X-ray diffraction patterns and XPS in detail (Supplementary Figs 11–16). The HER activities of Mo_2_C@NPC/NPRGO-1.1 and -3.3 were also evaluated using the same measurements. As seen from Fig. 5a,b, Mo_2_C@NPC/NPRGO showed the lowest onset overpotential and the smallest Tafel slope among the three samples. We speculate that these results are likely related to the amount and distribution of active sites. Because of the lower amount of Mo_2_C NPs in Mo_2_C@NPC/NPRGO-1.1, the corresponding electrocatalytic activity was poorer than that of Mo_2_C@NPC/NPRGO. In contrast, a larger number of Mo_2_C NPs in Mo_2_C@NPC/NPRGO-3.3 aggregated together, which is also unfavourable for the HER. These results demonstrate that the amount of PMo_12_ substantially influences the HER performance.

We subsequently studied the influence of carbonization temperature under the given conditions. Supplementary Figs 17–22 show the morphology, structure and composition of the two samples carbonized at 700 and 1,100 °C (defined as PMo_12_–PPy/RGO-700 and Mo_2_C@NPC/NPRGO-1100), respectively. The onset overpotentials of PMo_12_–PPy/RGO-700 and Mo_2_C@NPC/NPRGO-1100 were 20 and 27 mV, respectively, and the Tafel slopes were 48.4 and 70.1 mV dec^−1^, respectively (Fig. 5c and d). Among these catalysts, the Mo_2_C@NPC/NPRGO catalyst exhibited the optimal HER activity, possibly because active sites of Mo_2_C were not produced when PMo_12_–PPy/RGO is carbonized at 700 °C; the high–carbonization temperature led to substantial sintering and aggregation of Mo_2_C NPs, which further reduced the density of highly active sites. Meanwhile, the N content decreased with increasing carbonization temperature (Supplementary Table 1). All of these results were consistent with the SEM, TEM, XRD, thermogravimetric analysis and XPS results (Supplementary Figs 17–22). Therefore, in this work, the selection of the correct PMo_12_ content and carbonization temperature was critical to forming high-HER active sites.

### Theoretical investigation

**Figure 6:**
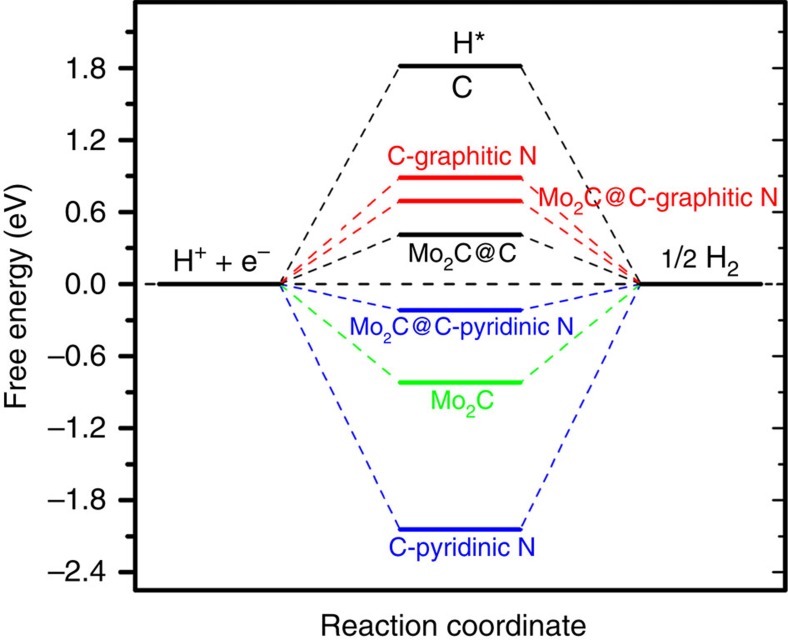
DFT-calculated HER activities. Calculated free energy diagram for HER on various studied system.

The aforementioned experimental results demonstrated that the Mo_2_C@NPC/NPRGO composite exhibits excellent electrocatalytic activity toward the HER because of the synergistic effects of Mo_2_C and NPC/NPRGO. To elucidate the mechanism underlying the superior HER activity of the Mo_2_C@NPC/NPRGO composite, we performed a series of DFT calculations (Supplementary Fig. 23 and Supplementary Table 3). Theoretically, the HER pathway can be depicted as a three-state diagram containing an initial state of H^+^ + *e*^−^, an intermediate state of adsorbed H (H*, where * denotes an adsorption site), and a final state of 1/2 the H_2_ product^5^,^22^. Generally, a good hydrogen evolution catalyst should have a free energy of adsorbed H of approximately zero (Δ*G*_H*_≈0), which can provide a fast proton/electron-transfer step as well as a fast hydrogen release process^42^. Because only trace amounts of P were present in the Mo_2_C@NPC/NPRGO hybrid compared to the N content, we investigated only the effect of N doping (graphitic N and pyridinic N) on the catalytic effect of the hybrids. Figure 6 shows the calculated free energy diagram for the HER in various studied systems.

According to our computational results, pristine graphene had an endothermic Δ*G*_H*_ of 1.82 eV, implying an energetically unfavourable interaction with hydrogen. Therefore, the HER can barely proceed on pristine graphene because of the slow proton/electron transfer. On the other hand, the (001) surface of Mo_2_C had a strong interaction with H, as indicated by the exothermic Δ*G*_H*_ of −0.82 eV, which would subsequently lead to poor HER performance because of the foreseeable difficulty of hydrogen release. Moreover, N-doped graphene exhibited low catalytic activity toward the HER. Specifically, the Δ*G*_H*_ values for graphitic-N- and pyridinic-N-doped graphene were 0.89 and −2.04 eV, respectively.

However, the catalytic activity of graphene and N-doped graphene were substantially improved when they were anchored to the surface of Mo_2_C. For example, the Δ*G*_H*_ values for Mo_2_C@C and Mo_2_C@C-graphitic N were 0.41 and 0.69 eV, respectively, which were much lower than those of suspended graphene (1.82 eV) and N-doped graphene (0.89 eV). The Δ*G*_H*_ of Mo_2_C@C (0.41 eV) indicated that the graphene C atoms in the hybrid also play an important role in the HER activity. In particular, due to the synergistic effect between Mo_2_C and C-pyridinic N, Mo_2_C@C-pyridinic N had a favourable Δ*G*_H*_ (−0.22 eV) for the adsorption and desorption of hydrogen. Therefore, the active sites for the HER should be composed mainly of pyridinic N atoms and C atoms of graphene rather than graphitic N atoms. We note here that, according to the results of XPS analysis, the major type of N in Mo_2_C@NPC/NPRGO was pyridinic N, which means that Mo_2_C@NPC/NPRGO would manifest a high density of active sites and would consequently present a high-current density at a low overpotential for the HER. Overall, the experimental and theoretical results verified that as-synthesized Mo_2_C@NPC/NPRGO is an unexpected and highly efficient HER electrocatalyst.

## Discussion

In view of the aforementioned considerations, the amazing HER activities of the Mo_2_C@NPC/NPRGO are postulated to originate from the following reasons: (1) the small size of Mo_2_C NPs favors the exposure of an abundance of available active sites, which may enhance the catalytic activity for the HER^7^,^21^,^28^; (2) the introduction of heteroatoms (N, P) into the carbon structure results in charge density distribution and asymmetry spin, thus enhancing the interaction with H^+^ (refs 18, 27). Especially, pyridinic N is favourable for highly efficient catalytic performance^43^,^44^; (3) as an advanced support, RGO can increase the dispersion of PMo_12_ to further obtain highly dispersed Mo_2_C during the carbonization process. Meanwhile, the outstanding electrical conductivity of RGO facilitates charge transfer in the catalyst^11^,^25^; (4) the robust conjugation between Mo_2_C and NPC/NPRGO provides a resistance-less path favourable for fast electron transfer. The carbon shells may hamper the aggregation of Mo_2_C NPs^21^ and promote electron penetration from Mo_2_C to RGO^22^. Furthermore, the geometric confinement of Mo_2_C inside the carbon shells can also enhance the catalytic activity for the HER^40^ and (5) the unique structure of Mo_2_C@NPC/NPRGO is favourable for the fast mass transport of reactants and facilitates electron transfer^26^,^39^. Because of the synergistic catalytic effects of the aforementioned factors, the Mo_2_C@NPC/NPRGO catalyst exhibits potent HER activity.

In summary, we designed and developed a novel architecture that is composed of Mo_2_C NPs, NPC and NPRGO by simply carbonizing a ternary PMo_12_–PPy/RGO nanocomposite. The effect of the PMo_12_ content and carbonization temperature on the HER activity was investigated in detail. The RGO-supported Mo-based catalysts prepared with PMo_12_–PPy/RGO as the precursor may efficiently hinder Mo sources and graphene from aggregating during the formation of RGO-supported Mo_2_C NPs. The obtained Mo_2_C@NPC/NPRGO nanocomposite exhibits the best HER performance and high stability as an electrocatalyst in an acidic electrolyte reported to date. Theoretical studies demonstrated that the synergistic effect between Mo_2_C and C-pyridinic N contributes to the excellent HER activity of the Mo_2_C@NPC/NPRGO nanocomposite, in accordance with the experimental results. This proof-of-concept study not only offers novel hydrogen-evolving electrocatalysts with excellent activity but also opens new avenues for the development of other 2D coupled nanohybrids with transition-metal carbides and RGO using POMs/conducting polymer/RGO as a precursor. These catalysts can also be explored as highly efficient electrocatalysts for oxygen reduction reaction (ORR), HER and lithium batteries.

## Methods

### Synthesis of PMo_12_–PPy/RGO and Mo_2_C@NPC/NPRGO hybrids

In a typical synthesis, GO NSs were pre-synthesized by chemical oxidation exfoliation of natural graphite flakes using a modified Hummers method^45^. The obtained GO NSs were dispersed in de-ionized water by ultrasonication to form a suspension with the concentration of 1 mg ml^−1^. Around 12.5 ml of such GO suspension and 150 ml of 2 mM PMo_12_ solution were added into a clean three-necked flask, respectively, and mixed uniformly under a strong ultrasonication bath. Subsequently, Py monomer solution by dispersing 230 μl of Py in 15 ml de-ionized water, was slowly dropped into the above mixed PMo_12_/GO suspension. With the addition of Py monomer solution, the reaction system gradually turned from yellow-brown to deep blue and a black precipitate began to generate after about 5 min. Finally, the reactor was transferred to an oil bath and allowed to react for 30 h at 50 °C under vigorously magnetic stirring. After separated by centrifugation and washed with deionized water and anhydrous ethanol for several times, the black PMo_12_–PPy/RGO ternary nanohybrids were obtained, which were dried in vacuum at 50 °C. In control experiments, PMo_12_–PPy/RGO (1.1) and PMo_12_–PPy/RGO (3.3) were synthesized by identical condition except that the amount of PMo_12_ is 1.1 and 3.3 g, respectively.

To prepare the Mo_2_C@NPC/NPRGO nanocomposite, 2 g of PMo_12_–PPy/RGO was carbonized in a flow of ultrapure N_2_ at 900 °C for 2 h with the heating rate of 5 °C min^−1^. The obtained samples were acid etched in H_2_SO_4_ (0.5 M) for 24 h with continuous agitation at 80 °C to remove unstable and inactive species. The etched samples were then thoroughly washed with de-ionized water until reaching a neutral pH, and defined as Mo_2_C@NPC/NPRGO, Mo_2_C@NPC/NPRGO-1.1 and -3.3, respectively.

### Synthesis of PMo_12_–PPy and Mo_2_C@NPC composites

The synthetic procedure is very similar to PMo_12_–PPy/RGO without GO. Likewise, the preparation of Mo_2_C@NPC composite is identical with that of Mo_2_C@NPC/NPRGO.

### Characterization

The TEM and HRTEM images were recorded on JEOL-2100F apparatus at an accelerating voltage of 200 kV. Surface morphologies of the carbon materials were examined by a SEM (JSM-7600F) at an acceleration voltage of 10 kV. The EDX was taken on JSM-5160LV-Vantage-typed energy spectrometer. The XRD patterns were recorded on a D/max 2500VL/PC diffractometer (Japan) equipped with graphite monochromatized Cu Kα radiation (*λ*=1.54060 Å). Corresponding work voltage and current is 40 kV and 100 mA, respectively. XPS was recorded by a scanning X-ray microprobe (PHI 5000 Verasa, ULAC-PHI, Inc.) using Al Kα radiation and the C1*s* peak at 284.8 eV as internal standard. The Raman spectra of dried samples were obtained on Lab-RAM HR800 with excitation by an argon ion laser (514.5 nm). The nitrogen adsorption–desorption experiments were operated at 77 K on a Micromeritics ASAP 2050 system. BET surface areas were determined over a relative pressure range of 0.05–0.3, during which the BET plot is linear. The pore size distributions were measured by using the nonlocalized density functional theory method. Before the measurement, the samples were degassed at 150 °C for 10 h.

### Electrochemical measurements

All electrochemical experiments were conducted on a CHI 760D electrochemical station (Shanghai Chenhua Co., China) in a standard three electrode cell in 0.5 M H_2_SO_4_ at room temperature. A glassy carbon electrode (3 mm in diameter), an Ag/AgCl with saturated KCl, and a Pt wire were used as the working electrode, reference and counter electrode, respectively. A total of 4 mg of the catalysts were dispersed in 2 ml of 9:1 v/v water/Nafion by sonication to form a homogeneous ink. Typically, 5 μl well-dispersed catalysts were covered on the glassy carbon electrode and then dried in an ambient environment for measurements. The electrocatalyst was prepared with a catalyst loading of 0.14 mg cm^−2^. Commercial 20% Pt–C catalyst was also used as a reference sample. Linear sweep voltammetry was tested with a scan rate of 5 mV s^−1^. EIS measurements were carried out from 1,000 kHz to 100 mHz with an amplitude of 10 mV at the open-circuit voltage. The electrochemical stability of the catalyst was conducted by cycling the potential between −0.3 and 0.3 V vs RHE at a scan rate of 100 mV s^−1^. The Chronoamperometry were tested at an overpotential of −0.12 V vs RHE after equilibrium. To estimate the electrochemical active surface areas of the catalysts, CV was tested by measuring EDLC under the potential window of 0.19–0.39 vs RHE with various scan rate (20, 40, 60, 80, 100, 120, 140, 160, 180 and 200 mV s^−1^). A flow of N_2_ was maintained over the electrolyte during the experiment to eliminate dissolved oxygen. The potential vs RHE was converted to RHE via the Nernst equation: *E*_RHE_=*E*_Ag/AgCl_+0.059pH+*E*^θ^_Ag/AgCl_. In 0.5 M H_2_SO_4_, *E*_RHE_=0.21 V+*E*_Ag/AgCl_.

### Computational details

DFT calculations were performed using the plane-wave technique implemented in the Vienna *ab initio* Simulation package^46^. The ion–electron interaction was treated within the projector-augmented plane wave pseudopotentials^47^,^48^. The generalized gradient approximation expressed by Perdew−Burke−Ernzerhof functional^49^ and a plane-wave cutoff energy of 360 eV were used in all computations. The electronic structure calculations were employed with a Fermi-level smearing of 0.1 eV for all surface calculations and 0.01 eV for all gas-phase species. The Brillouin zone was sampled with 3 × 3 × 1 *k*-points. The convergence of energy and forces were set to 1 × 10^−5^ eV and 0.02 eV Å^−1^, respectively. A vacuum region of around 12 Å was set along the *z* direction to avoid the interaction between periodic images. More computational details are provided in Supplementary Note 1.

## Additional information

**How to cite this article**: Li, J.-S. *et al*. Coupled Molybdenum Carbide and Reduced Graphene Oxide Electrocatalysts for Efficient Hydrogen Evolution. *Nat. Commun.* 7:11204 doi: 10.1038/ncomms11204 (2016).

## Supplementary Material

Supplementary Information Supplementary Figures 1-23, Supplementary Tables 1-3, Supplementary Note 1 and Supplementary References

## Figures and Tables

**Table 1:** Comparison of catalytic parameters of different HER catalysts.

**Catalyst**	**Onset potential (mV** **vs** **RHE)**	**Overpotential at 10 mA cm**^−**2**^ **(mV** **vs** **RHE)**	***j***_**0**_ **(mA cm**^−**2**^**)**	**Tafel slope (mV dec**^−**1**^**)**
Mo_2_C@NPC	137	260	3.16 × 10^−3^	126.4
Mo_2_C@NPC/NPRGO	0	34	1.09	33.6
Pt–C	0	40	0.39	30

HER, H_2_ evolution reaction; Pt–C, 20 wt% Pt on carbon lack from Johnson–Matthey; RHE, reversible hydrogen electrode.

*j*_0_ represents exchange current density that was calculated from Tafel curves using extrapolation method.
