# Supplementary material for: Coupled molybdenum carbide and reduced graphene oxide electrocatalysts for efficient hydrogen evolution
Source: Nat Commun. 2016 Apr 1;7:11204. doi: 10.1038/ncomms11204 (PMC4822009; doi:10.1038/ncomms11204)
Supplement: Supplementary Information — Supplementary Figures 1-23, Supplementary Tables 1-3, Supplementary Note 1 and Supplementary References [file ncomms11204-s1.pdf]

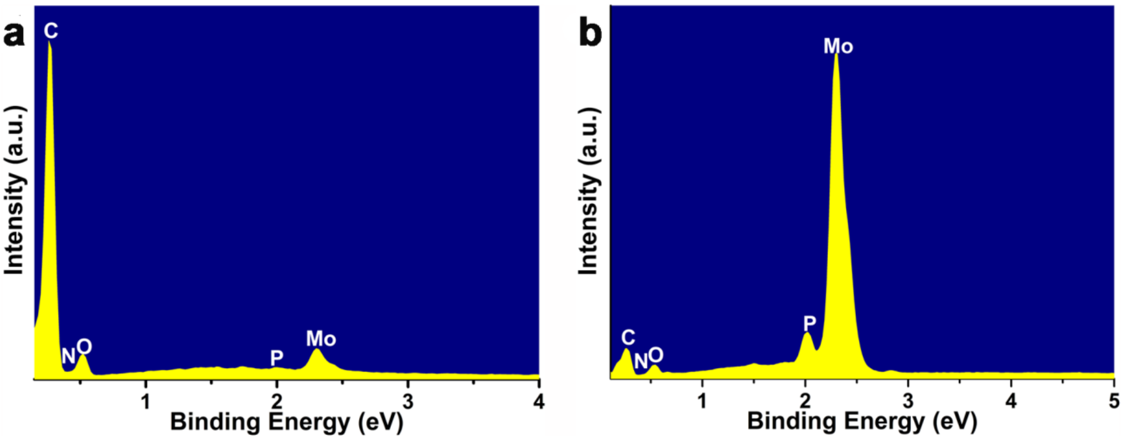

Supplementary Figure 1. (**a-b**) EDX of Mo_2C@NPC/NPRGO and Mo_2C@NPC.

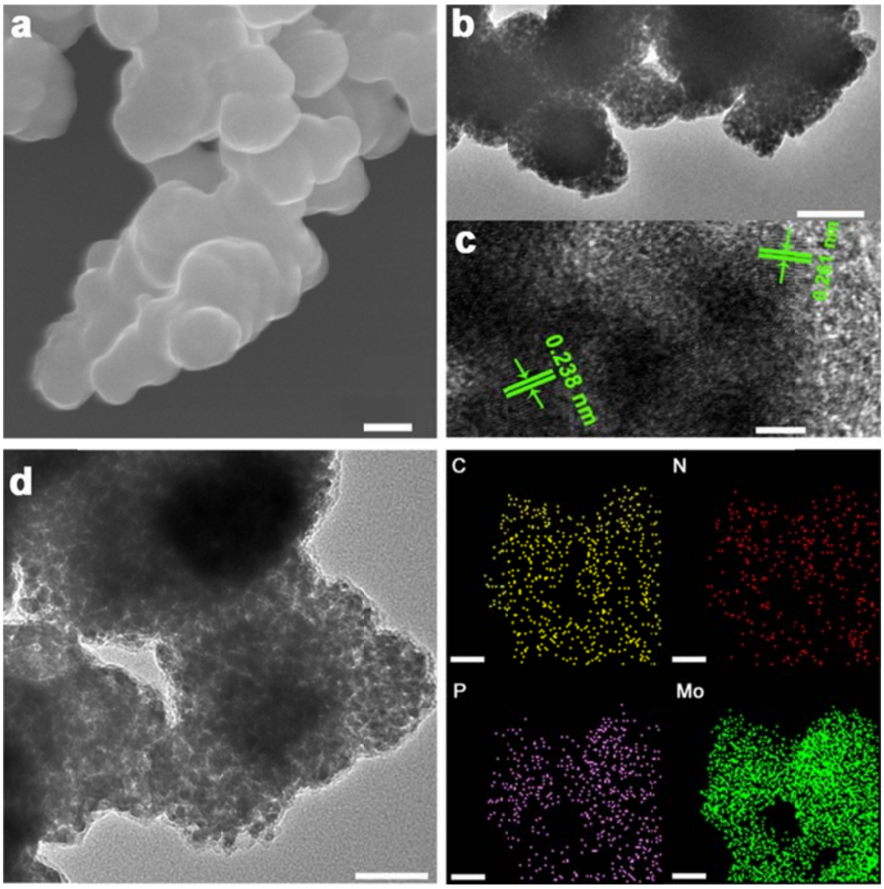

Supplementary Figure 2. (**a**) SEM image of PMo_{12}-PPy, (**b**) TEM, (**c**) HRTEM, (**d**) STEM image and EDX elemental mapping of C, N, P, and Mo of Mo_2C@NPC. Scale bar: **a** (200 nm); **b** (100 nm); **c** (5 nm); **d** (50 nm).

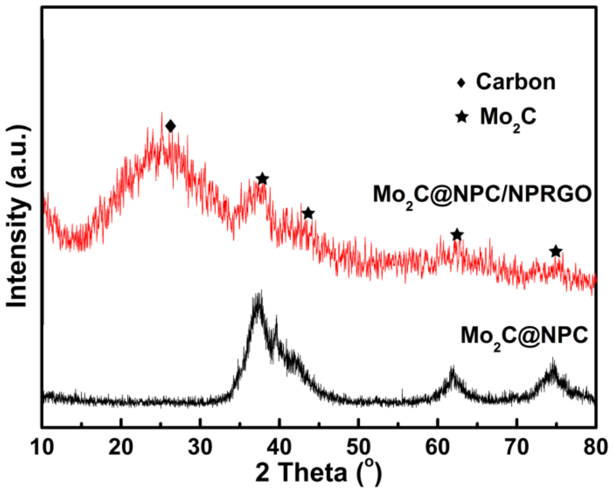

Supplementary Figure 3. PXRD patterns of Mo_2C@NPC and Mo_2C@NPC/NPRGO.

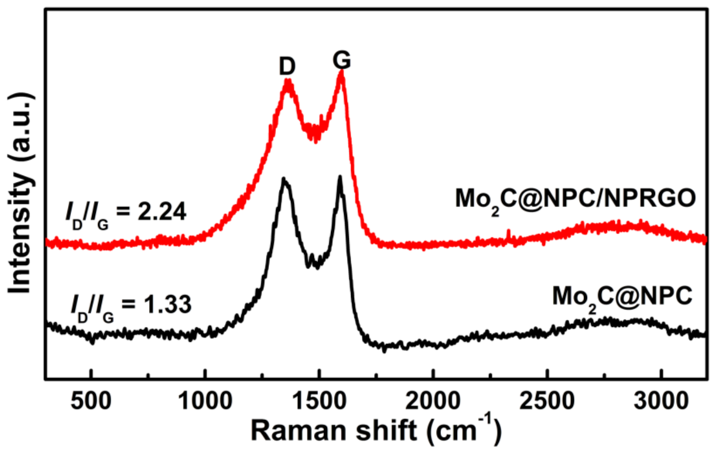

Supplementary Figure 4. Raman spectra of Mo_2C@NPC and Mo_2C@NPC/NPRGO.

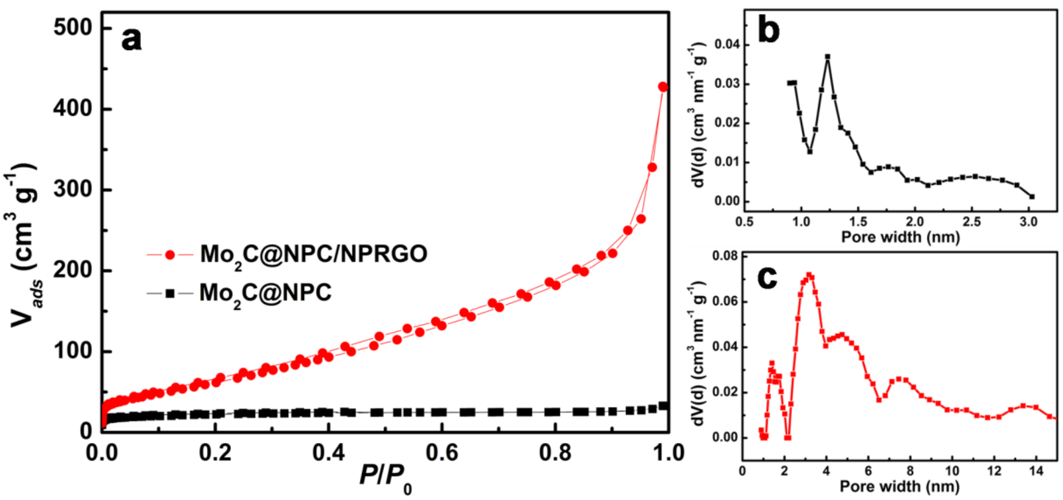

Supplementary Figure 5. (**a**) N_2 sorption isotherms of Mo_2C@NPC and Mo_2C@NPC/NPRGO, (**b-c**) the corresponding pore size distribution by NLDFT method.

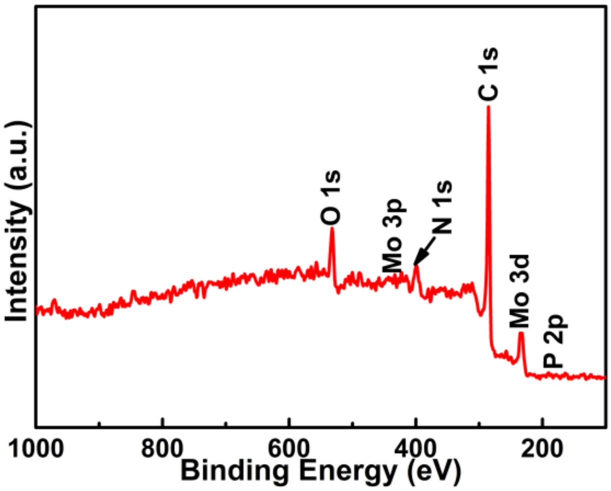

Supplementary Figure 6. XPS survey spectrum of Mo_2C@NPC/NPRGO.

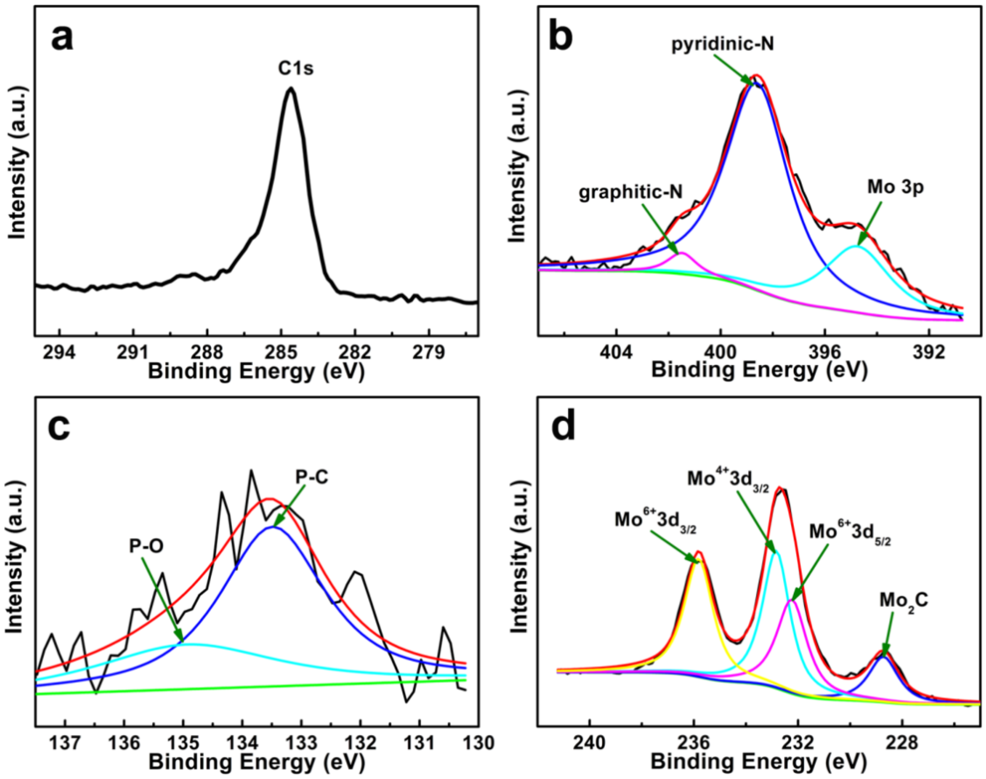

Supplementary Figure 7. XPS high-resolution scans of (**a**) C 1s, (**b**) N 1s, (**c**) P 2p, (**d**) Mo 3d electrons of Mo_2C@NPC.

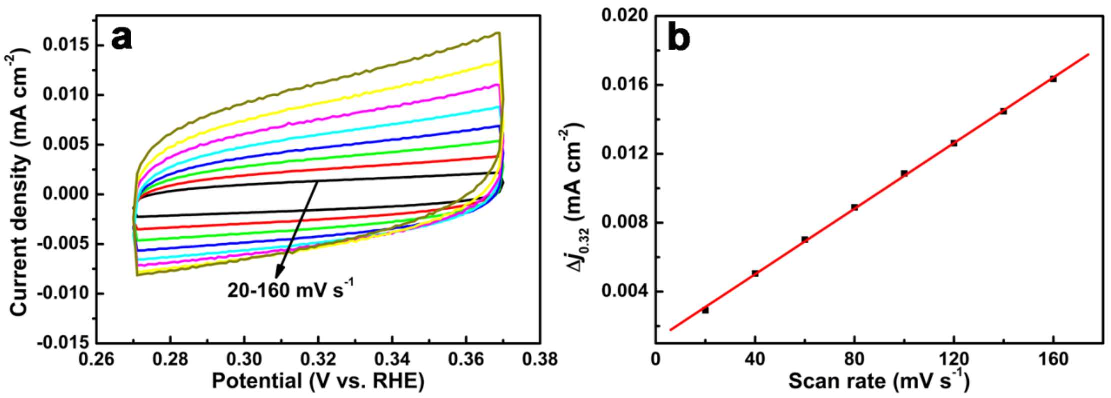

Supplementary Figure 8. (**a**) CVs of Mo_2C@NPC with different rates from 20 to 160 mV s^{-1}. (**b**) The capacitive current at 0.32 V as a function of scan rate for Mo_2C@NPC.

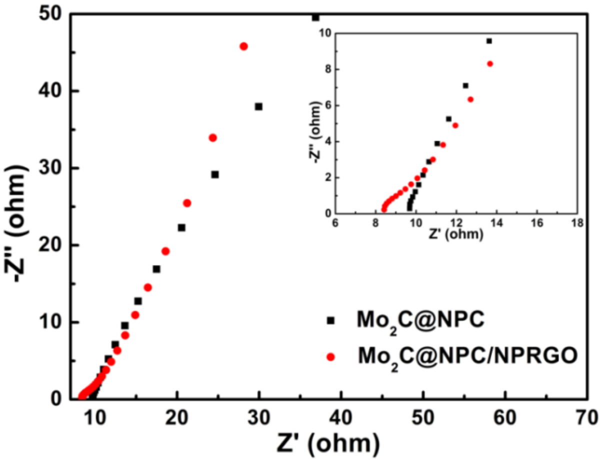

Supplementary Figure 9. Electrochemical impedance spectra (EIS) of three electrocatalysts over the frequency ranging from 1000 kHz to 0.1 Hz at the open-circuit voltage. Inset denotes the magnified images of high frequency region.

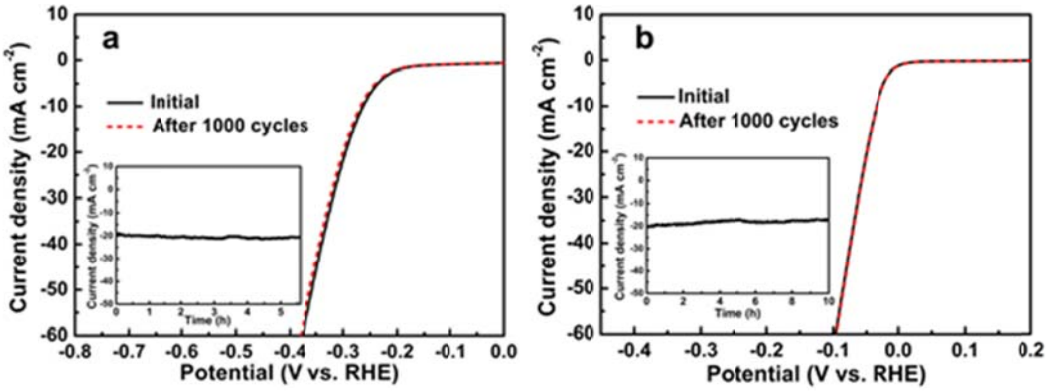

Supplementary Figure 10. (**a-b**) Polarization curves of Mo_2C@NPC and Mo_2C@NPC/NPRGO initially and after 1000 cycles, respectively. (**a-b**) Inset: Time-dependent current density curve of Mo_2C@NPC and Mo_2C@NPC/NPRGO under a static overpotential of 296 and 48 mv, respectively.

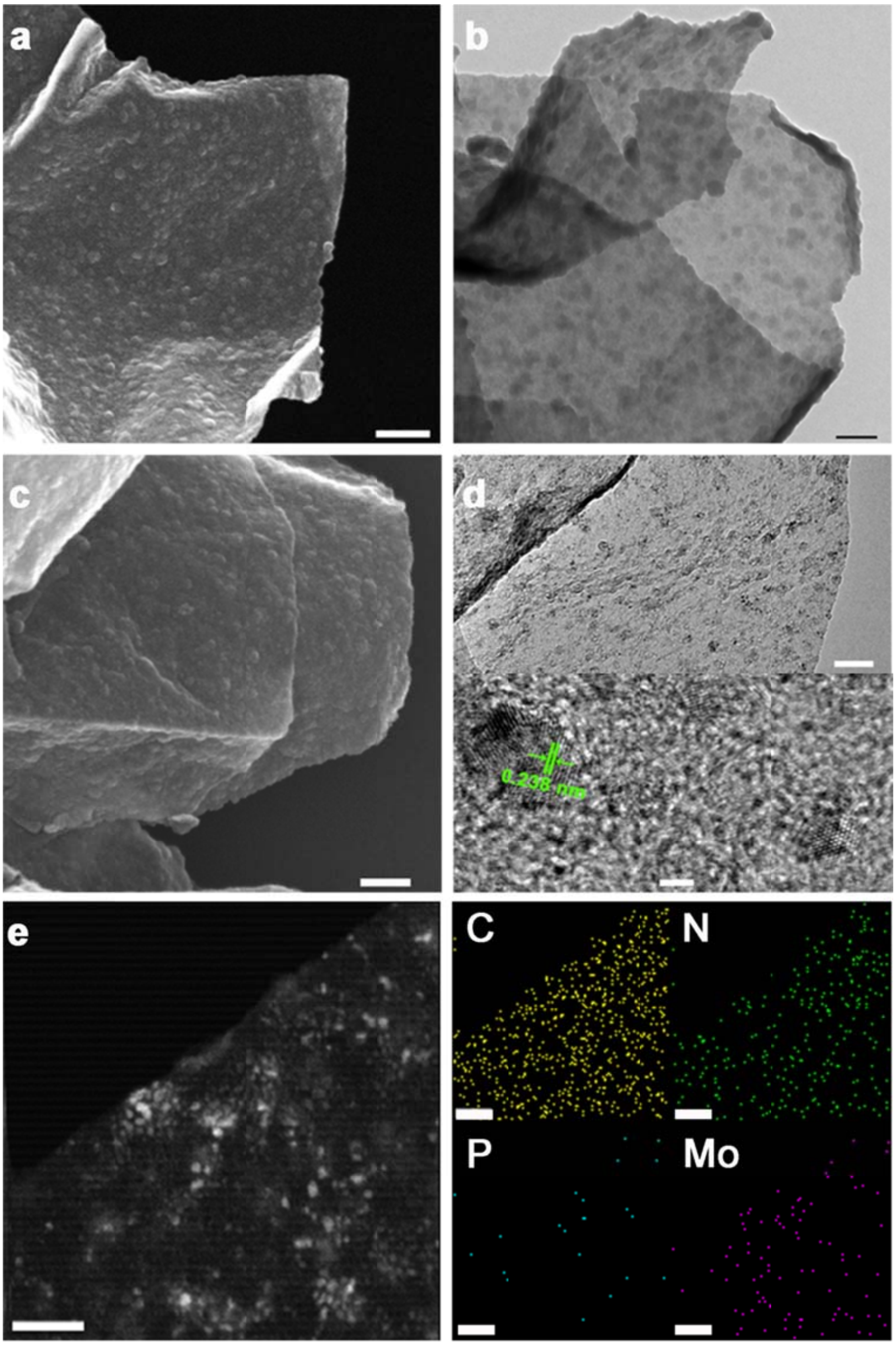

Supplementary Figure 11. (**a**) SEM and (**b**) TEM images of PMo_{12}-PPy/RGO (1.1), (**c**) SEM, (**d**) TEM (inset: HRTEM), (**e**) STEM image and EDX elemental mapping of C, N, P, and Mo of Mo_2C@NPC/NPRGO (1.1). Scale bar: **a** (200 nm); **b, c** (100 nm); **d** (100 and 2 nm); **e** (50 nm).

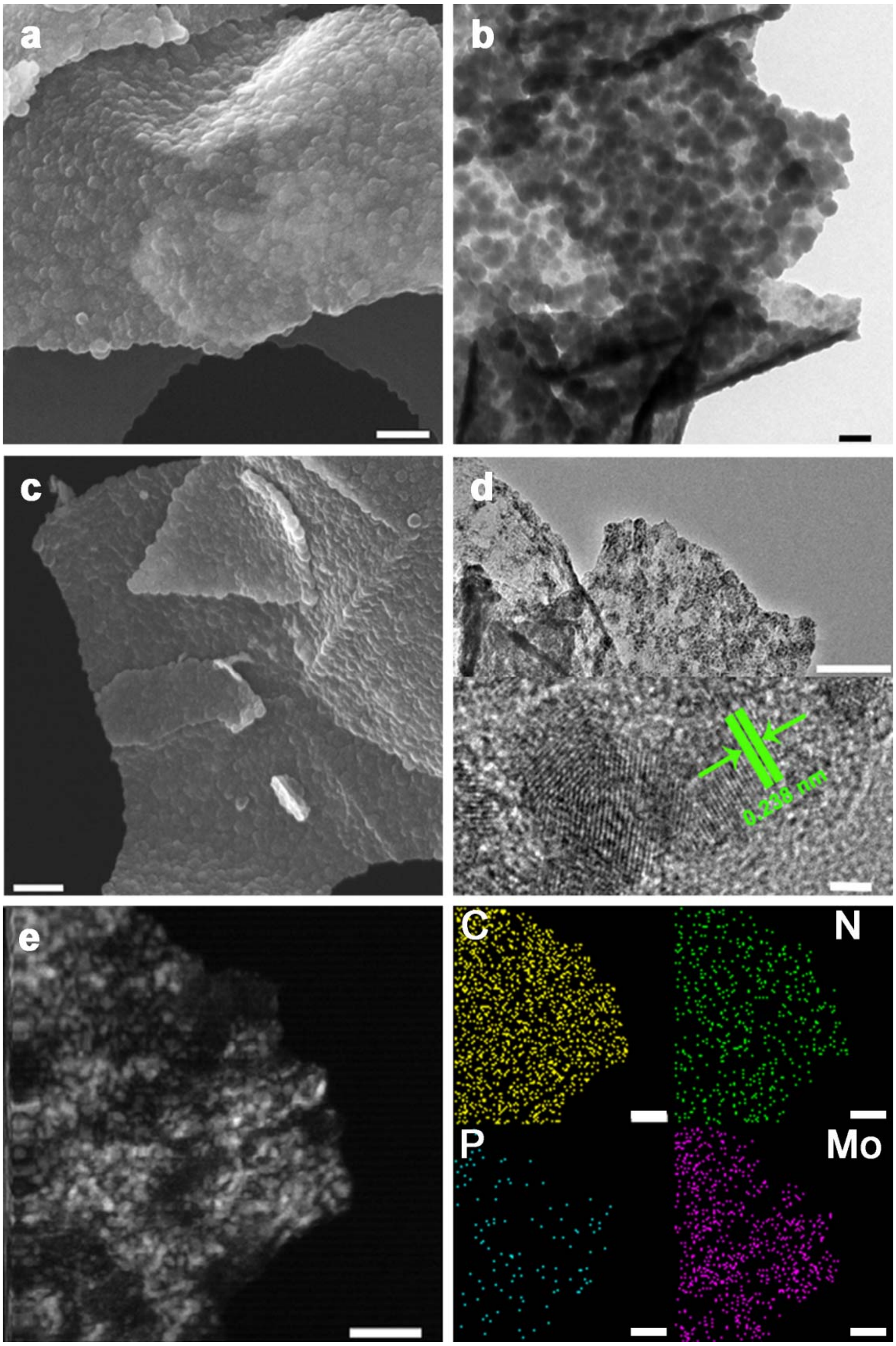

Supplementary Figure 12. (**a**) SEM and (**b**) TEM images of PMo_{12}-PPy/RGO (3.3), (**c**) SEM, (**d**) TEM, (inset: HRTEM), (**e**) STEM image and EDX elemental mapping of C, N, P, and Mo of Mo_2C@NPC/NPRGO (3.3). Scale bar: **a** (200 nm); **b** (100 nm); **c** (200 nm); **d** (200 and 2 nm); **e** (50 nm).

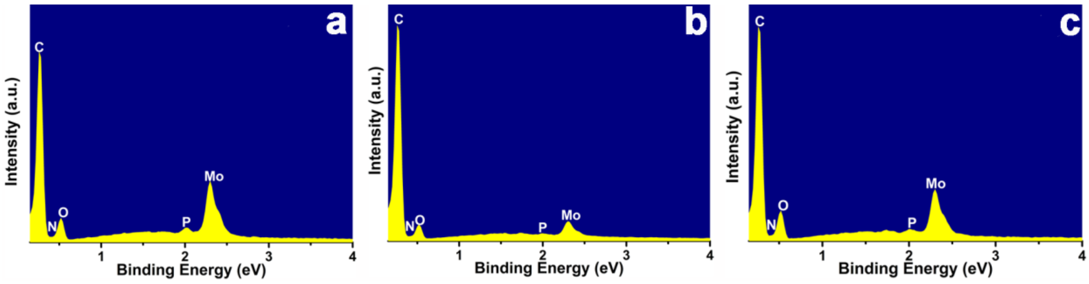

Supplementary Figure 13. (**a-c**) EDX patterns of Mo_2C@NPC/NPRGO (1.1, 2.2, and 3.3).

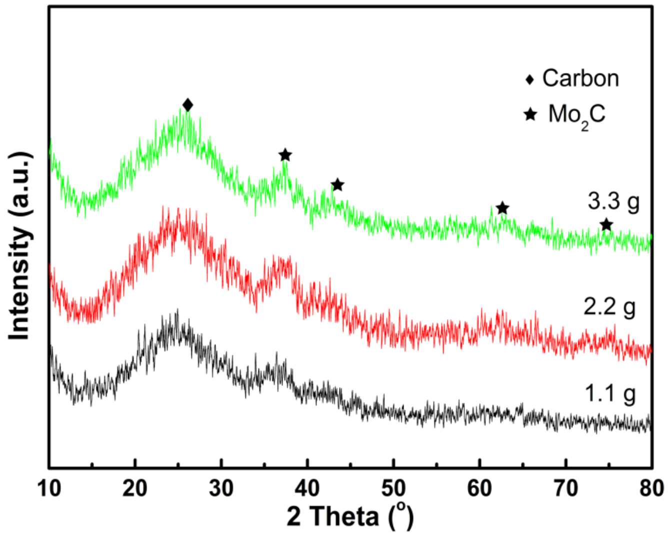

Supplementary Figure 14. PXRD patterns of Mo_2C@NPC/NPRGO (1.1, 2.2, and 3.3).

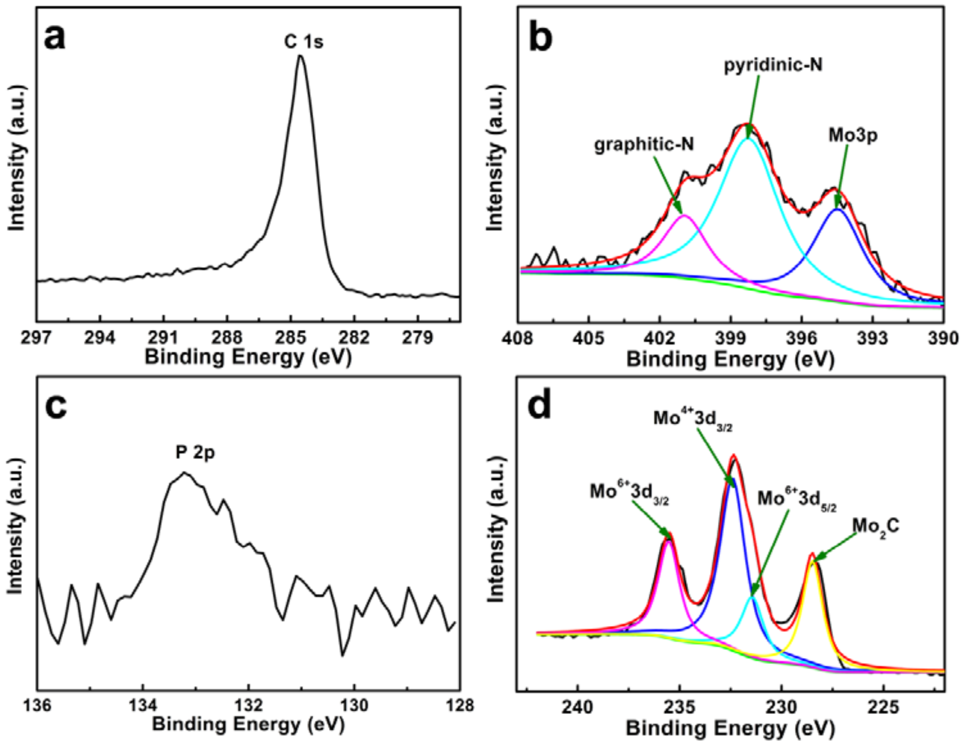

Supplementary Figure 15. XPS high-resolution scans of (**a**) C 1s, (**b**) N 1s, (**c**) P 2p, (**d**) Mo 3d electrons of Mo_2C@NPC/NPRGO (1.1).

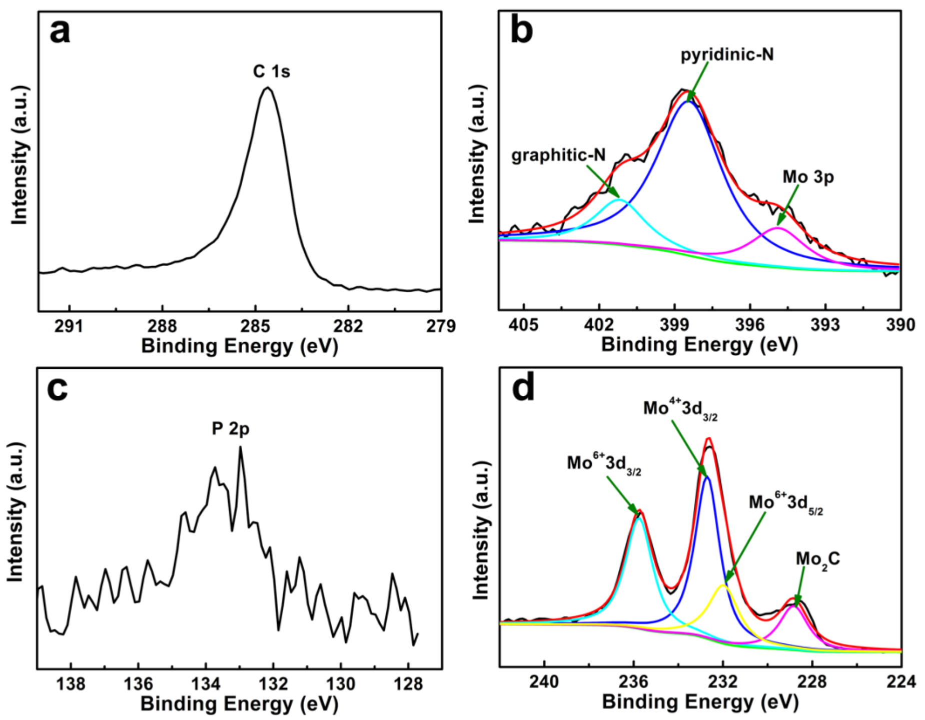

Supplementary Figure 16. XPS high-resolution scans of (**a**) C 1s, (**b**) N 1s, (**c**) P 2p, (**d**) Mo 3d electrons of Mo_2C@NPC/NPRGO (3.3).

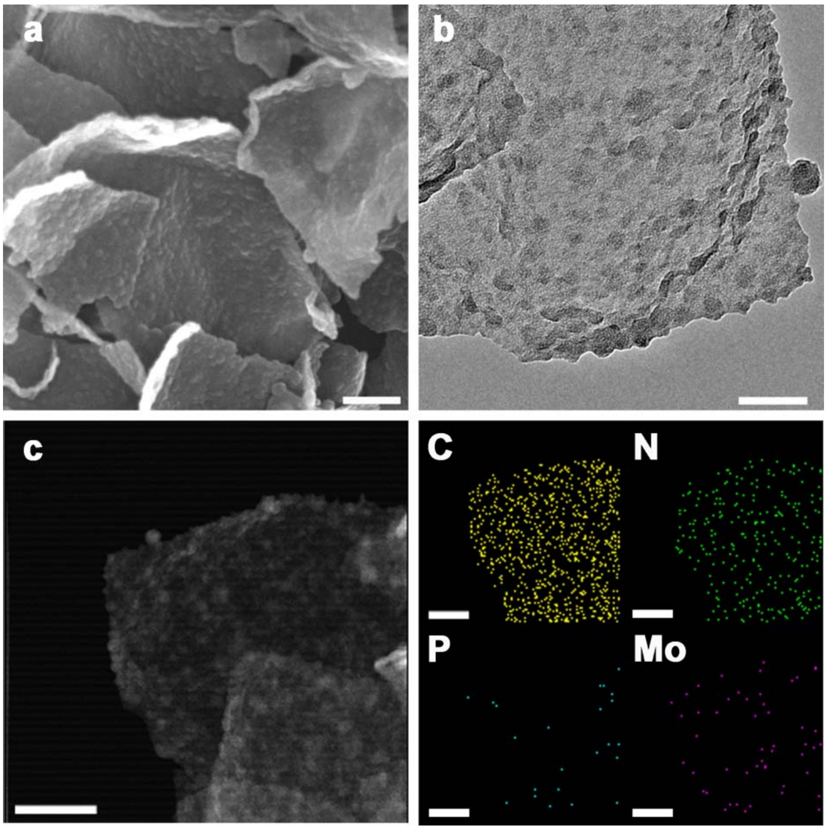

Supplementary Figure 17. (**a**) SEM, (**b**) TEM, (**c**) STEM image and EDX elemental mapping of C, N, P, and Mo of PPy-PMo_{12}/RGO-700. Scale bar: **a** (200 nm); **b** (100 nm); **c** (200 nm).

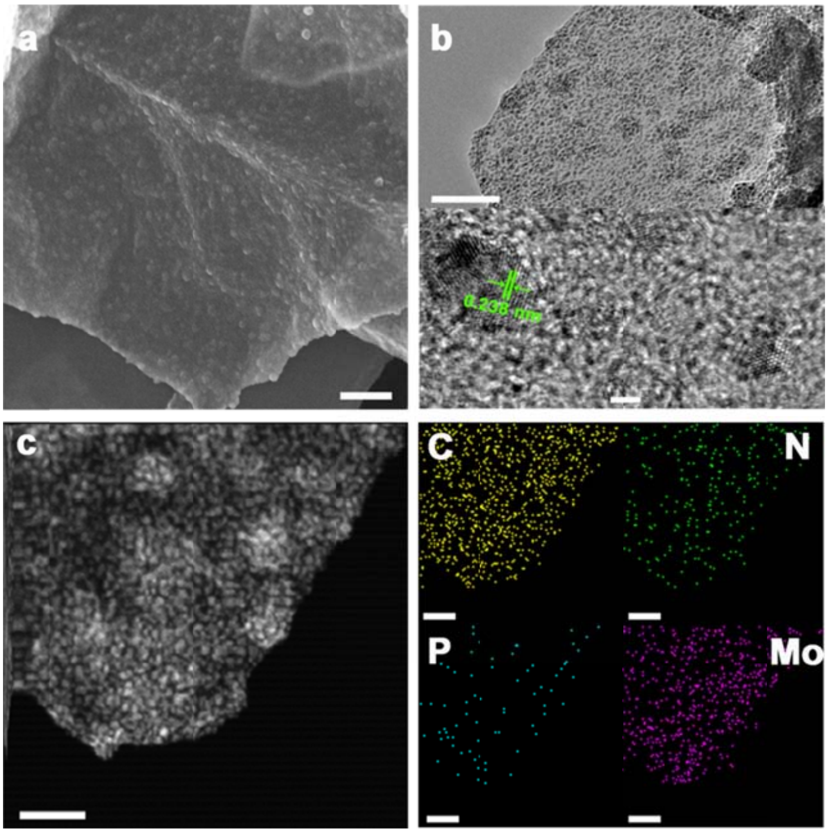

Supplementary Figure 18. (**a**) SEM, (**b**) TEM (inset: HRTEM), (**c**) STEM image and EDX elemental mapping of C, N, P, and Mo of Mo_2C@NPC/NPRGO-1100. Scale bar: **a** (200 nm); **b** (100 and 2 nm); **c** (50 nm).

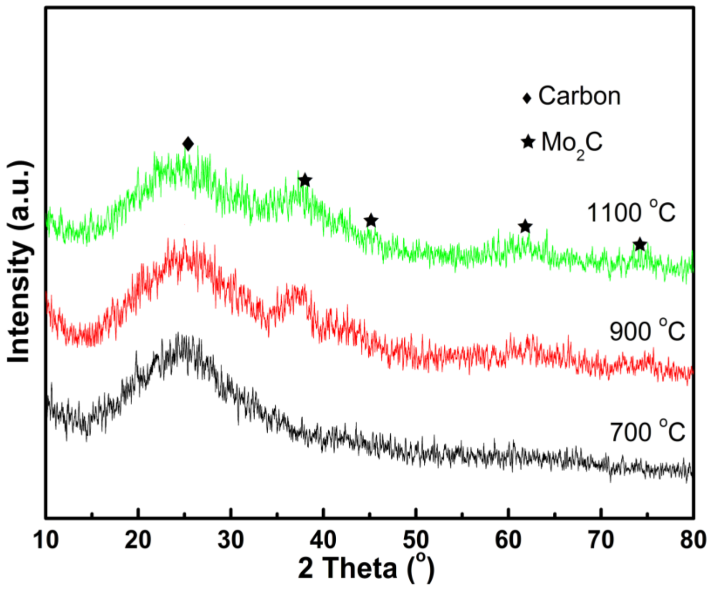

Supplementary Figure 19. PXRD of PMo_{12}-PPy/RGO-700, Mo_2C@NPC/NPRGO, and Mo_2C@NPC/NPRGO-1100.

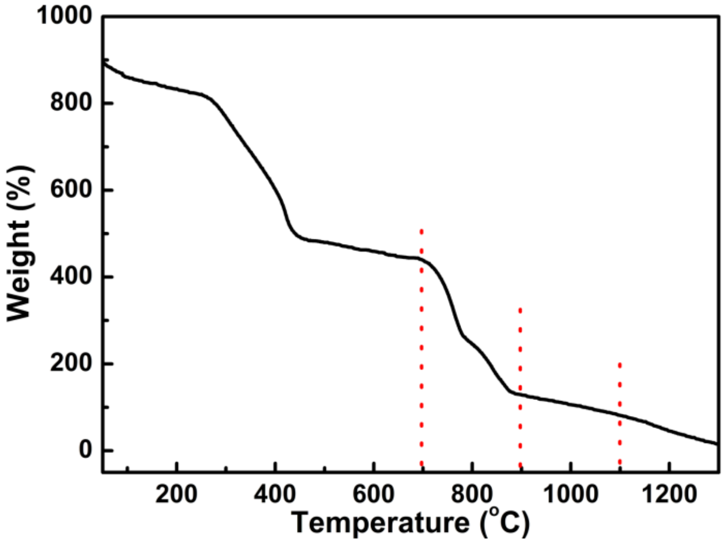

Supplementary Figure 20. TG of PMo_{12}-PPy/RGO.

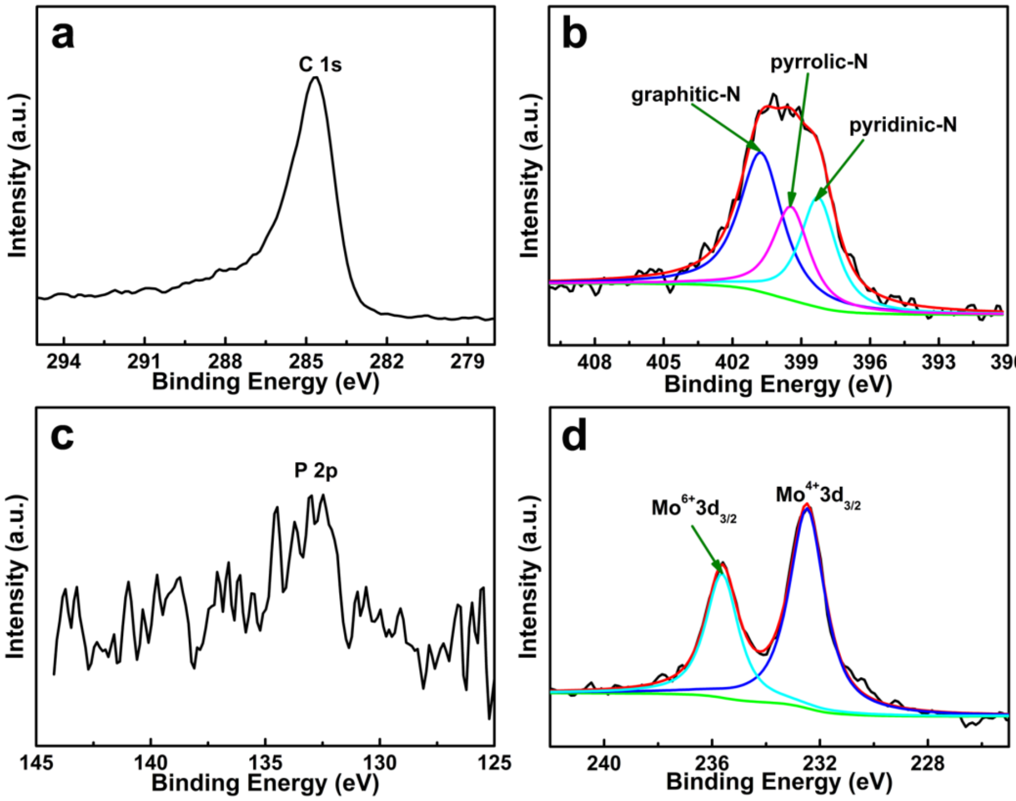

Supplementary Figure 21. XPS high-resolution scans of (**a**) C 1s, (**b**) N 1s, (**c**) P 2p, (**d**) Mo 3d electrons of PMo_{12}-PPy/RGO-700.

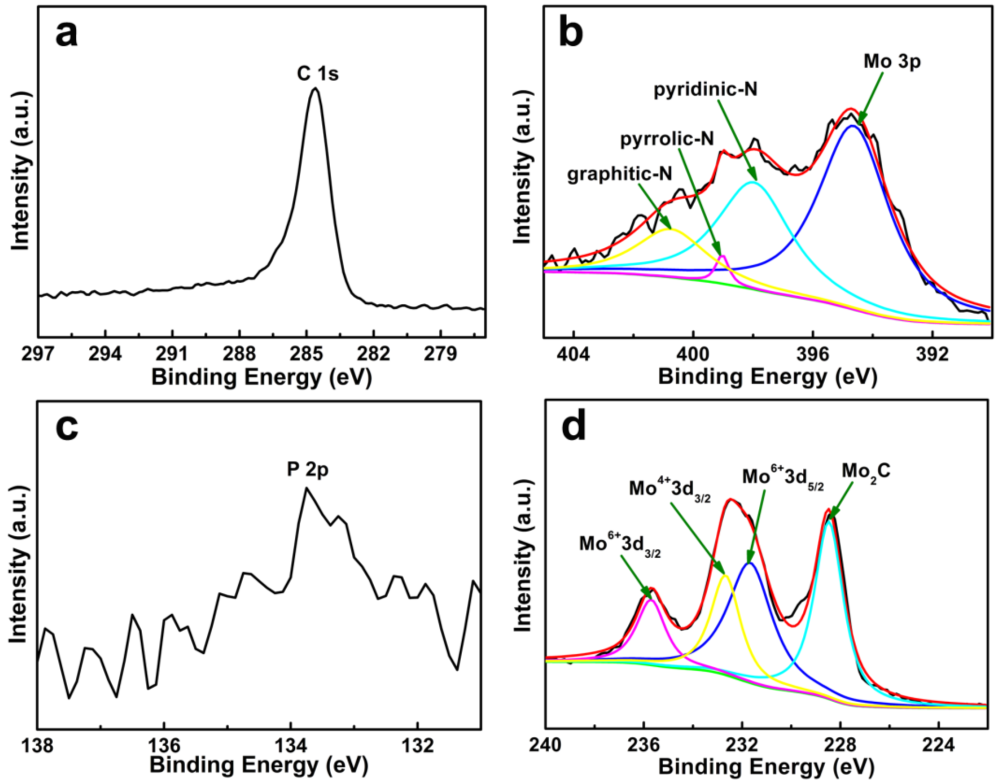

Supplementary Figure 22. XPS high-resolution scans of (**a**) C 1s, (**b**) N 1s, (**c**) P 2p, (**d**) Mo 3d electrons of Mo_2C@NPC/NPRGO-1100.

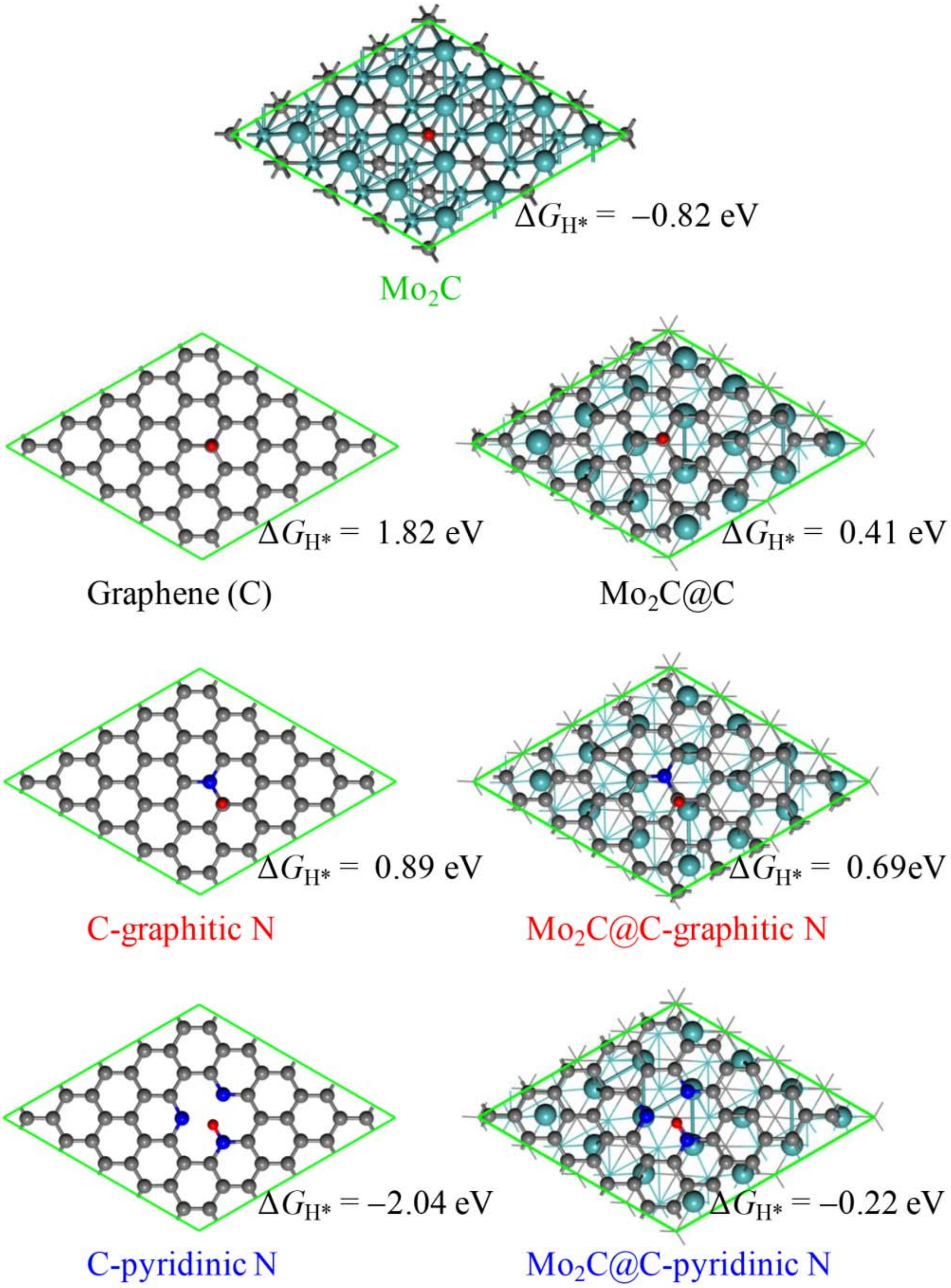

Supplementary Figure 23. The theoretical models of the studied systems. The gray, blue, cyan, and red balls represent C, N, Mo, and H atoms, respectively.

Supplementary Table 1. Atomic percents of different catalysts by XPS measurement.

catalyst	C (at. %)	N (at. %)	O (at. %)	P (at. %)	Mo (at. %)
Mo_2C@NPC	79.26	2.12	10.85	0.72	7.05
PMo_{12}-PPy/RGO-700	85.86	2.59	10.48	0.43	0.64
Mo_2C@NPC/NPRGO	87.76	1.82	7.53	0.39	2.5
Mo_2C@NPC/NPRGO-1100	88.26	1.31	7.72	0.28	2.43
Mo_2C@NPC/NPRGO-1.1	86.04	1.85	9.65	0.41	2.05
Mo_2C@NPC/NPRGO-3.3	85.7	1.76	9.34	0.44	2.76

Supplementary Table 2. Comparison of HER performance in acidic media for Mo_2C@NPC/NPRGO with other non-noble metal electrocatalysts.

Catalyst	Onset potential (mV vs RHE)	Tafel slope (mV dec^{-1})	J_0 (mA cm^{-2})[1]	Ref.
**Mo_2C@NPC/NPRGO**	**0**	**33.6**	**1.09**	**This work**
MoO_2@PC-RGO	0	41	0.48	1
MoCx	-25	53	0.023	2
MoDCA-5	-6	41	0.179	3
Mo_2C@NC	-50	60	_	4
Mo_2C/CNT-GR	-70	58	0.062	5
PDAP-MoCN-CO_2	-50	46	_	6
Mo_2C/GCSs	-120	62.6	0.0125	7
Mo_2C/CNT	0	65.3	0.014	8
β-Mo_2C	-50	120	0.0173	9
MoS_2/$CoSe_2$	-11	36	0.073	10
np-Mo_2C NW	-70	53	_	11
β-Mo_2C/reduced graphene oxide	-20	66.4	0.037	12
MWCMNs	-35	46	0.64	13
Mo_2C nanotubes	-82	62	0.017	14
NS-doped Mo_2C	-46	47	0.038	15
1T-VS_2	-20	34	_	16
$CoMoS_3$	-75	56.9	0.011	17
P-WN/rGO	-46	54	0.35	18
Fe-WCN	-100	47.1	_	19
Ni_2P nanoparticles	-20	46	0.033	20
CoP nanoparticles	-20	50	0.14	21
Cu_3PNW/CF	-62	67	0.18	22
CoNi@NC	0	104	_	23
PCN@N-graphene-750	-8	49.1	0.43	24
$MoS_{2.7}$@NPG	-18	41	_	25
FeP NA/Ti	-16	38	0.42	26
$NiMoN_x$/C	-78	35.9	0.24	27
WS_2 nanosheets	-100	70	_	28
$Fe_{0.9}Co_{0.1}S_2$/CNT	-90	46	_	29

Note: [1] represents the exchange current density.

Supplementary Table 3. The adsorption energy of H species (ΔE_{H*}), the relevant contributions to the free energy (E_{ZPE} and TS), and the free energy of adsorbed H (ΔG_{H*}) on different surfaces.

Species	ΔE_{H*}	E_{ZPE} (eV)	–TS (eV)	ΔG_{H*} (eV)
H_2	\	0.27	–0.41	\
H* on C	1.44	0.31	–0.001	1.82
H* on C-graphitic N	0.50	0.31	–0.001	0.89
H* on C-pyridinic N	–2.39	0.28	–0.002	–2.04
H* on Mo_2C	–1.06	0.18	–0.004	–0.82
H* on Mo_2C@C	0.032	0.31	–0.001	0.41
H* on Mo_2C@C-graphitic N	0.31	0.31	–0.001	0.69
H* on Mo_2C@C-pyridinic N	–0.56	0.28	–0.002	–0.22

Supplementary Note 1

Supplementary Figure 23 shows the theoretical models of the studied systems. For the structure model of hexagonal Mo_2C bulk, the calculated lattice parameters are a = b = 6.07 Å,

b = 6.07 Å, and c = 4.72 Å, which is in good agreement with the experimental value (a = b = 2 × 3.002 Å and c = 4.724 Å). Mo_2C (001) surface is modeled with six layers of atoms in Mo-termination. The model of (N-doped) graphene is constructed as 5 × 5 periodic supercell (a = b = 12.30 Å) comprising 50 C atoms. Since there is lattice mismatch between Mo_2C (001) and (N-doped) graphene, a 1.2% stretched 2 × 2 Mo_2C (001) supercell is employed to fit a 5 × 5 graphene supercell. In addition, for the systems that involve Mo_2C (001), the top four layers of Mo_2C (001) and graphene (plus adsorbate H) are allowed to relax, while the rest of the slab (the bottom two layers of Mo_2C (001)) remained fixed.

The free energy of adsorbed H (ΔG_{H*}) on different surfaces are calculated as:

$$\Delta G_{H*} = \Delta E_{H*} + \Delta E_{ZPE} - T\Delta S \quad (1)$$

where ΔE_{H*} is the adsorption energy of H species. ΔE_{ZPE} and ΔS are the energy change in zero point energy and entropy, respectively. T is the system temperature (298.15 K, in our work). For H* on different surfaces, all 3N degrees of freedom are treated as vibrational motions while neglecting the contributions from the material surfaces. ZPE and S are calculated from temperature, pressure and calculated vibrational energy by using standard methods.[30] Therefore, ΔE_{ZPE} can be computed by $\Delta E_{ZPE} = E_{ZPE\text{-}H*} - 1/2E_{ZPE\text{-}H2}$ and ΔS can be obtained by $\Delta S = S_{H*} - 1/2S_{H2}$. The calculated $E_{ZPE\text{-}H2}$ value is 0.271 eV and S_{H2} is the entropy of H_2 in the gas phase at standard conditions. Finally, ΔG_{H*}, for instance, in pristine graphene (C), is calculated to be ΔE_{H*} + 0.378 eV, which is well approaching to pervious HER calculation of carbon materials (ΔE_{H*} + 0.37 eV).[4] The adsorption energy of H species ΔE_{H*}, the relevant contributions to the free energy, and free energy of adsorbed H ΔG G_{H*} are summarized in Supplementary Table 3.

Supplementary References

1. Tang Y. J. *et al.* Porous molybdenum-based hybrid catalysts for highly efficient hydrogen evolution. *Angew. Chem. Int. Ed.* **54**, 12928-12932 (2015).

2. Wu H. B., Xia B. Y., Yu L., Yu X. Y. & Lou X. W. Porous molybdenum carbide nano-octahedrons synthesized via confined carburization in metal-organic frameworks for efficient hydrogen production. *Nat. Commun.* **6**, 6512 (2015).

3. Ma R. *et al.* Ultrafine molybdenum carbide nanoparticles composited with carbon as a highly active hydrogen-evolution electrocatalyst. *Angew. Chem. Int. Ed.* **54**, 14723-14727 (2015).

4. Liu Y. *et al.* Coupling Mo_2C with nitrogen-rich nanocarbon leads to efficient hydrogen-evolution electrocatalytic sites. *Angew. Chem. Int. Ed.* **54**, 10752-10757 (2015).

5. Youn D. H. *et al.* Highly active and stable hydrogen evolution electrocatalysts based on molybdenum compounds on carbon nanotube–graphene hybrid support. *ACS Nano* **8**, 5164-5173 (2014).

6. Zhao Y., Kamiya K., Hashimoto K. & Nakanishi S. In situ CO_2-emission assisted synthesis of molybdenum carbonitride nanomaterial as hydrogen evolution electrocatalyst. *J. Am. Chem. Soc.* **137**, 110-113 (2015).

7. Cui W. *et al.* Mo_2C nanoparticles decorated graphitic carbon sheets: biopolymer-derived solid-state synthesis and application as an efficient electrocatalyst for hydrogen generation. *ACS Catal.* **4**, 2658-2661 (2014).

8. Chen W. F. *et al.* Highly active and durable nanostructured molybdenum carbide electrocatalysts for hydrogen production. *Energy Environ. Sci.* **6**, 943-951 (2013).

9. Wan C., Regmi Y. N. & Leonard B. M. Multiple phases of molybdenum carbide as electrocatalysts for the hydrogen evolution reaction. *Energy Environ. Sci.* **53**, 6407-6410 (2014).

10. Gao M. R. *et al.* An efficient molybdenum disulfide/cobalt diselenide hybrid catalyst for electrochemical hydrogen generation. *Nat. Commun.* **6**, 5982 (2015).

11. Liao L. *et al.* A nanoporous molybdenum carbide nanowire as an electrocatalyst for hydrogen evolution reaction. *Energy Environ. Sci.* **7**, 387-392 (2014).

12. Chen W. F. *et al.* Biomass-derived electrocatalytic composites for hydrogen evolution. *Energy Environ. Sci.* **6**, 1818-1826 (2013).

13. Wu R., Zhang J., Shi Y., Liu D. & Zhang B. Metallic WO_2–carbon mesoporous nanowires as highly efficient electrocatalysts for hydrogen evolution reaction. *J. Am. Chem. Soc.* **137**, 6983-6986 (2015).

14. Ma F. X., Wu H. B., Xia B. Y., Xu C.Y. & Lou X. W. Hierarchical β-Mo_2C nanotubes organized by ultrathin nanosheets as a highly efficient electrocatalyst for hydrogen

production. *Angew. Chem. Int. Ed.* **54**, 15395-15399 (2015).

15. Ang H*. et al.* Hydrophilic nitrogen and sulfur Co-doped molybdenum carbide nanosheets for electrochemical hydrogen evolution. *Small*, **11**, 6278-6284 (2015).

16. Yuan J*. et al.* Facile synthesis of single crystal vanadium disulfide nanosheets by chemical vapor Deposition for efficient Hydrogen evolution reaction. *Adv. Mater.* **27**, 5605-5609 (2015).

17. Yu L., Xia B. Y., Wang X. & Lou XW. General formation of M–MoS_3 (M = Co, Ni) hollow structures with enhanced electrocatalytic activity for hydrogen evolution. *Adv. Mater*. **28**, 92-97 (2016).

18. Yan H*, et al.* Phosphorus-modified tungsten nitride/reduced graphene oxide as a high-performance, non-noble-metal electrocatalyst for the hydrogen evolution reaction. *Angew. Chem. Int. Ed.* **54**, 6325-6329 (2015).

19. Zhao Y., Kamiya K., Hashimoto K. & Nakanishi S. Hydrogen evolution by tungsten carbonitride nanoelectrocatalysts synthesized by the formation of a tungsten acid/polymer hybrid in Ssitu. *Angew. Chem. Int. Ed.* **52**, 13638-13641 (2013).

20. Popczun E. J*., et al.* Nanostructured nickel phosphide as an electrocatalyst for the hydrogen evolution reaction. *J. Am. Chem. Soc.* **135**, 9267-9270 (2013).

21. Popczun E. J., Read C. G., Roske C. W., Lewis N. S. & Schaak R. E. Highly Active Electrocatalysis of the Hydrogen Evolution Reaction by Cobalt Phosphide Nanoparticles. *Angew. Chem. Int. Ed.* **53**, 5427-5430 (2014).

22. Tian J., Liu Q., Cheng N., Asiri A. M. & Sun X. Self-supported Cu_3P nanowire arrays as an Iintegrated high-performance three-dimensional cathode for generating hydrogen from water. *Angew. Chem. Int. Ed.* **53**, 9577-9581 (2014).

23. Deng J., Ren P., Deng D. & Bao X. Enhanced electron penetration through an ultrathin graphene layer for highly efficient catalysis of the hydrogen evolution reaction. *Angew. Chem. Int. Ed.* **54**, 2100-2104 (2015).

24. Duan J., Chen S., Jaroniec M. & Qiao S. Z. Porous C_3N_4 nanolayers@N-graphene films as catalyst electrodes for highly efficient hydrogen evolution. *ACS Nano* **9**, 931-940 (2015).

25. Ge X., *et al*. Nanoporous metal enhanced catalytic activities of amorphous molybdenum sulfide for high-efficiency hydrogen production. *Adv. Mater.* **26**, 3100-3104 (2014).

26. Jiang P., *et al*. A cost-effective 3D hydrogen evolution cathode with high catalytic activity: FeP nanowire array as the active phase. *Angew. Chem. Int. Ed.* **53**, 12855–12859 (2014).

27. Chen W-F*, et al.* Hydrogen-evolution catalysts based on non-noble metal nickel–molybdenum nitride nanosheets. *Angew. Chem. Int. Ed.* **51**, 6131-6135 (2012).

28. Lukowski MA*, et al.* Highly active hydrogen evolution catalysis from metallic WS_2

nanosheets. *Energy Environ. Sci.* **7**, 2608-2613 (2014).
29. Wang D-Y*, et al.* Highly active and stable hybrid catalyst of cobalt-doped FeS2 nanosheets–carbon nanotubes for hydrogen evolution reaction. *J. Am. Chem. Soc.* **137***,* 1587−1592 (2015).
30. Cramer, C. J. Essentials of Computational Chemistry Theories and Models, 2004, Vol. 42, pp. 334–342.
